# A new 10-D hyperchaotic system with coexisting attractors and high fractal dimension: Its dynamical analysis, synchronization and circuit design

**DOI:** 10.1371/journal.pone.0266053

**Published:** 2022-04-12

**Authors:** Khaled Benkouider, Toufik Bouden, Aceng Sambas, Badis Lekouaghet, Mohamad Afendee Mohamed, Sulaiman Ibrahim Mohammed, Mustafa Mamat, Mohd Asrul Hery Ibrahim, Muhammad Zaini Ahmad

**Affiliations:** 1 Automatic Department, University of MSB Jijel, Ouled Aissa, Jijel, Algeria; 2 Department of Mechanical Engineering, Universitas Muhammadiyah Tasikmalaya, Tasikmalaya, West Java, Indonesia; 3 Faculty of Informatics and Computing, Universiti Sultan Zainal Abidin, Kuala Terengganu, Malaysia; 4 School of Quantitative Sciences, College of Art and Sciences, Universiti Utara Malaysia, Sintok, Kedah, Malaysia; 5 Institute of Strategic Industrial Decision Modelling (ISIDM), School of Quantitative Sciences, Universiti Utara Malaysia, UUM Sintok, Kedah, Malaysia; 6 Faculty of Entrepreneurship and Business, Universiti Malaysia Kelantan (UMK), Kota Bharu, Kelantan, Malaysia; 7 Institute of Engineering Mathematics, Universiti Malaysia Perlis, Kuala Perlis, Arau, Perlis, Malaysia; Instituto Nacional de Astrofisica Optica y Electronica, MEXICO

## Abstract

This work introduce a new high dimensional 10-D hyperchaotic system with high complexity and many of coexisting attractors. With the adjustment of its parameters and initial points, the novel system can generate periodic, quasi-periodic, chaotic, and hyperchaotic behaviours. For special values of parameters, we show that the proposed 10-D system has a very high Kaplan-Yorke fractal dimension, which can reach up to 9.067 indicating the very complexity of the 10-D system dynamics. In addition, the proposed system is shown to exhibit at least six varied attractors for the same values of parameters due to its multistability. Regions of multistability are identified by analysing the bifurcation diagrams of the proposed model versus its parameters and for six different values of initial points. Many of numerical plots are given to show the appearance of different dynamical behaviours and the existence of multiple coexisting attractors. The main problem with controlling chaos/hyperchaos systems is that they are not always fully synchronized. therefore, some powerful synchronization techniques should be considered. The synchronization between the high-dimensional 10-D system and a set of three low-dimensional chaotic and hyperchaotic systems is proposed. Ten control functions are designed using the active control method, ensuring synchronisation between the collection of systems and the 10-D hyperchaotic system. Finally, using Multisim 13.0 software to construct the new system’s electronic circuit, the feasibility of the new system with its extremely complicated dynamics is verified. Therefore, the novel 10-D hyperchaotic system can be applied to different chaotic-based application due to its large dimension, complex dynamics, and simple circuit architecture.

## Introduction

Scientific communities have been interested in chaotic systems study over the past 60 years, especially since the work of Edward Lorenz, the famous American meteorologist in 1963 [1]. The essential trait of chaotic systems, he discovered, is their great sensitivity to initial conditions. A small change in the chaotic system’s initial parameters results in significantly varied and unpredictable behaviour. This type of system’s tremendous complexity makes it beneficial in a variety of fields, including secure communication [2–5].

The Kaplan-Yorke dimension and the Lyapunov exponents are the most important tools for describing chaotic behaviour in a dynamical system [6]. Kaplan-Yorke dimension, on the other hand, is an effective measure of the fractal dimension and chaotic complexity of the normal n-dimensional dynamical system, and it is calculated using Lyapunov exponent values. When calculating the Lyapunov exponent, the dynamical system’s two adjacent starting values are taken into account. The paths produced via the initial guesses will exponentially diverge if this system exhibits chaotic behaviour, and the coefficients that characterises the divergence rate is a Lyapunov exponent. There is, absolutely, a Lyapunov exponent for every state-space dimension. At least one of the exponents must be positive for a dynamic system to display chaotic behavior. When there are many non-negative exponents, the related systems’ dynamics expand in multiple directions, resulting in a more complex behaviour, which we name a hyperchaotic system in this situation.

Many papers have been published on hyperchaotic system. Vaidyanathan et al. [7] proposed of the new 4-D hyperchaotic system with no equilibrium and analysis of global hyperchaos synchronization results of the new hyperchaotic system using Integral Sliding Mode Control (ISMC). Singh et al. [8] proposed of the 5-D hyperchaotic system with stable equilibrium point and the proposed system exhibits multistability and transient chaotic behavior. Alattas et al. [9] proposed of the synchronization problem of hyperchaotic systems using integral-type sliding mode control for the 6-D hyperchaotic systems and presented of the analog electronic circuit using MultiSIM. Lagmiri et al. [10] constructed of the two new 7D hyperchaotic systems and to investigate the dynamics and synchronization of these new systems using the theory of observers. Kang et al. [11] proposed a color image encryption method combining with 2D-VMD and 8D hyperchaotic system. Zhu et al [12] presented a nine-dimensional eight-order chaotic system, and the corresponding circuit implementation. Mahmoud et al. [13] presented another complex nonlinear hyperchaotic model, spoke to by nine first-order nonlinear ordinary differential equations and proposed new nine-dimensional chaotic Lorenz System with quaternion variables [14]. Jianliang et al. [15] proposed a ten-dimensional nine-order chaotic system and the electronic circuit implementation. However, there is still a need for discovering systems with different 10D hyperchaotic system.

Synchronization of chaotic systems has attracted much attention in recent years due to their applications in neuron model, robotic and cryprosystem. Yu et al. [16] presented a novel 5D hyperchaotic four-wing memristive system with multiline equilibrium and synchronization of the 5D hyperchaotic system with different structures by active control. Zambrano-Serrano and Anzo-Hernández [17] proposed a novel chaotic oscillator derived from the generic four-dimensional autonomous jerk systems and analyze the synchronization behavior of the chaotic oscillator via feedback control. Munoz-Pacheco et al. [18] analyzed the effect of a non-local fractional operator in an asymmetrical glucose-insulin regulatory system and proposed an active control scheme for forcing the chaotic regime (an illness) to follow a periodic oscillatory state, i.e., a disorder-free equilibrium. However, to the best of the authors’ knowledge, neither the control nor the synchronisation of the new 10-D hyperchaotic system has been investigated yet with the active control method.

Secure transmissions utilising various methods and schemes is one of chaotic system’s most essential applications. Chaotic systems generate complex signals with a random appearance, which are used to conceal the secret information to be communicated. As a result, many literature have studied the chaotic systems, so as to address the huge gap for the type of complicated system in the disciplines of chaotic encryption and secure communication [19, 20]. Nazari et al. [21] proposed secure transmission of authenticated medical images using a novel chaotic IWT-LSB blind watermarking approach. design an embedded cryptosystem based on a pseudo-random number generator (PRNG). Trujillo-Toledo et al. [22] proposed design an embedded cryptosystem based on a pseudo-random number generator (PRNG)using enhanced sequences from the Logistic 1D map, and it reaches a throughput of up to 47.44 Mbit/s using a personal computer with a 2.9 GHz clock, and 10.53 Mbit/s using a Raspberry Pi 4. Hemdan [23] presented a medical image watermarking approach based on Wavelet Fusion (WF), Singular Value Decomposition (SVD), and Multi-Level Discrete Wavelet Transform (M-DWT) with scrambling techniques for securing the watermarks images. García-Guerrero et al. [24] introduces a process to improve the randomness of five chaotic maps that are implemented on a PIC-microcontroller. They have improved chaotic maps tested to encrypt digital images in a wireless communication scheme, particularly on a machine-to-machine (M2M) link, via ZigBee channels. Silva-Juárez et al. [25] proposed the use of first-order all-pass and low-pass filters to design the ratio of the polynomials that approximate the fractional-order. Also, the filters are implemented using amplifiers and synthesized on a field-programmable analog array (FPAA) device. Tlelo-Cuautle et al. [26] provides guidelines to implement fractional-order derivatives using commercially available devices and describes details on using FPGAs to approach fractional-order chaotic systems, programming in VHDL and reducing hardware resources.

In addition, as previously stated, several studies discovered that the hyperchaotic systems with high dimensional (*n* > 3) whose positive Lyapunov exponent is more than one and having a high Kaplan-Yorke dimension is capable of generating more random and complex signals with greater uncertainty, which improves the chaotic transmissions security. Based of these reasons, several types of these high dimensional systems have been developed having two positive Lyapunov exponents since after the emergence the first system by Rossler in 1979 [27]. Some nonlinear dynamical systems can develop many forms of complexity such as chaos, hyperchaos, bifurcation and multistability. A dynamical system that generate two or more synchronize different attractors for a given set of coefficients is defined to be multistable.

In the recent years, construction new high dimensional (*n* > 5) hyperchaotic systems with high fractal dimension [28] and extreme multistability become an interesting area of research in chaos theory because of the need of these kinds of hyper-complex systems in recent engineering applications especially in secure communications. In this work, we generate the first 10-D hyperchaotic system which exhibit up to six synchronize attractors having high Kaplan-Yorke fractal dimension. The new 10-D hyperchaos system’s dynamic properties is discussed, its Regions of multistability identified, its active control synchronization and design its equivalent electronic circuit described.

The novelty and contributions of the paper are summarised as follows:

1. System has four positive parameters, twenty-three terms with two quadratic and one quartic nonlinearity.
2. This work reports various types of complexity behaviors in 10D hyperchaotic system, such as Chaos, Hyperchaos and Quasi-Periodic.
3. System has multistability, i.e. coexistence of chaotic attractors under various conditions.
4. System has unstable and self-excited family.
5. This work studied the synchronization of the proposed 10D system with three diverse Hyperchaotic and chaotic systems via active controllers.
6. The equivalent electronic circuit for the new 10-D hyperchaotic system (1) is developed using Multisim 13.0 software.

The rest of this paper is organized as follows. Section 2 describes the dynamics of the new 10D Hyperchaotic system. Dynamical analyses of the new 10D Hyperchaotic system are shown in Section 3. multistability and coexisting attractors in the new 10D Hyperchaotic system is discussed in Section 4. In Section 5 we discuss the synchronization of the new 10D hyperchaotic systems using active control. Circuit implementation of the new 10-D hyperchaotic system are presented in Section 6. Finally, the conclusions of this paper are summarized in Section 7.

## New 10-D hyperchaotic system

There are four positive parameters in the new 10D hyperchaotic system, as well as twenty-three terms with two quadratic and one quartic nonlinearity. The new system is describe using the algebraic equations (1):

$$\begin{array}{c} \left\{ \begin{array}{c} \dot{x_{1}}=x_{3}+x_{1}x_{2}-x_{1},\\ \dot{x_{2}}=1+a(x_{2}-x_{1}^{4})-x_{1}^{2},\\ \dot{x_{3}}=-x_{1}+x_{3}+x_{4},\\ \dot{x_{4}}=-bx_{3}+cx_{5},\\ \dot{x_{5}}=-x_{4}+x_{6},\\ \dot{x_{6}}=-x_{5}+x_{7},\\ \dot{x_{7}}=-x_{6}+x_{8},\\ \dot{x_{8}}=-x_{7}+(1-d)x_{9},\\ \dot{x_{9}}=-x_{8}+x_{10},\\ \dot{x_{10}}=-x_{9}+x_{7}. \end{array}\right. \end{array}$$ (1)

where the state variables are given as *x*_1_, *x*_2_, *x*_3_, *x*_4_, *x*_5_, *x*_6_, *x*_7_, *x*_8_, *x*_9_ and *x*_10_ while *a*, *b*, *c* and *d* parameters denote the positive constant. When the initial guess are selected as:

$$\begin{array}{c} (1,0,0,0,0,0,0,0,0,0). \end{array}$$ (2)

and the coefficient values are selected as:

$$\begin{array}{c} a=0.1,\;b=0.1,\;c=1.1,\;d=0.01. \end{array}$$ (3)

**Fig 1:**
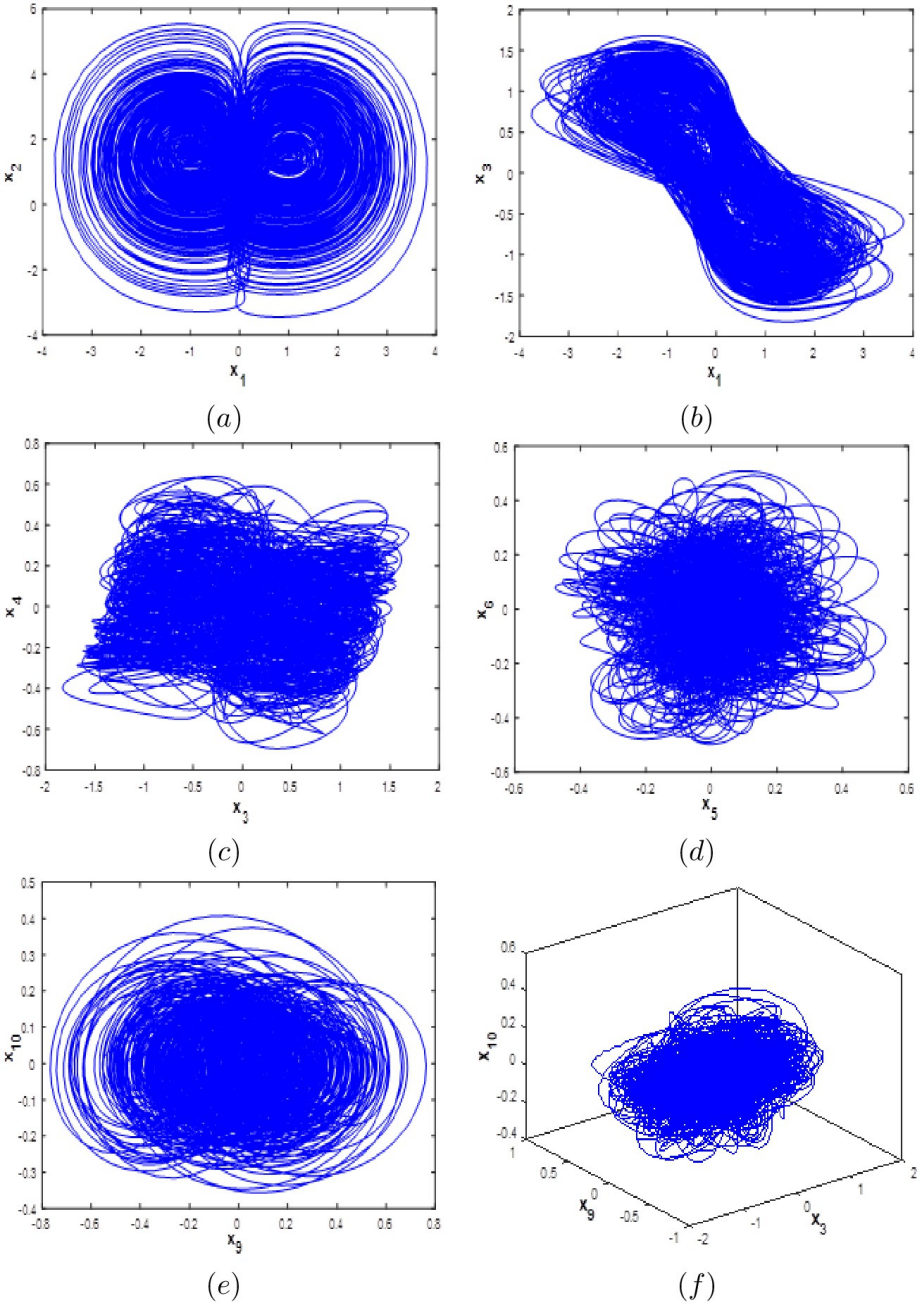
Phase portraits of the 10-D hyperchaotic system (1): (a) *x*_1_ − *x*_2_ attractor, (b) *x*_1_ − *x*_3_ attractor, (c) *x*_3_ − *x*_4_, (d) *x*_5_ − *x*_6_ attractor, (e) *x*_9_ − *x*_10_ attractor and (f) *x*_3_ − *x*_9_ − *x*_10_ attractor.

System (1) exhibit a complex hyperchaotic behavior with high fractal dimension and its phase portraits are described in Fig 1 using the Matlab ode45 function. It clear from the Fig 1 that our proposed the new 10D hyperchaotic system generates two-wing attractors.

**Fig 2:**
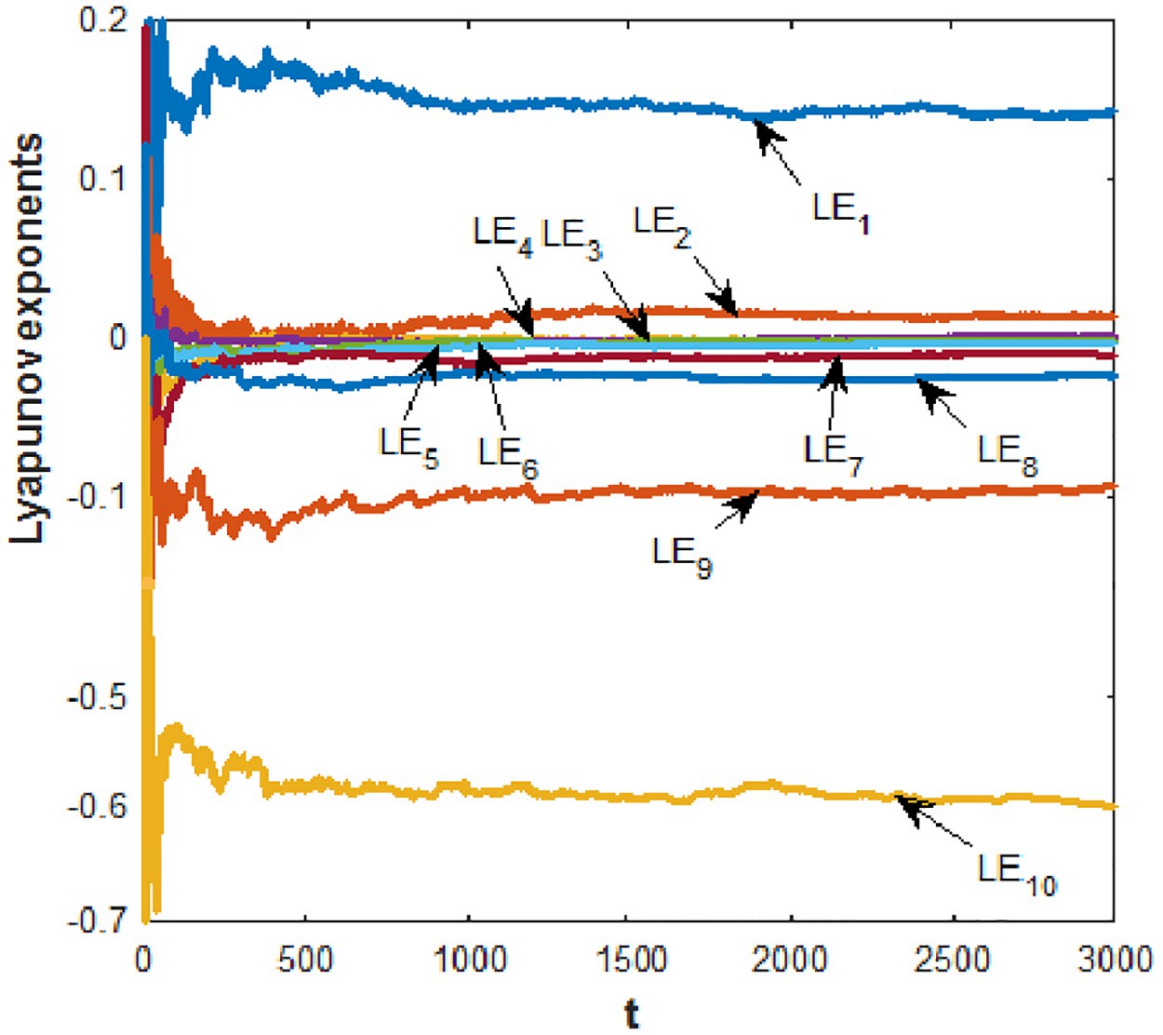
Lyapunov exponents of the new 10-D hyperchaotic system (1) with a = 0.1, b = 0.1, c = 1.1 and d = 0.01.

The Lyapunov exponents (*LE*) for the new 10D hyperchaotic system (1) whose initial conditions is given in Eq (2) and the parameters values as in Eq (3) can be calculated using Wolf’s algorithm, results are shown in Fig 2.

The obtained ten *LE* of the new 10D hyperchaotic system (1) are:

$$\begin{array}{c} \left\{ \begin{array}{c} LE_{1}=0.142,\\ LE_{2}=0.038,\\ LE_{3}=0,\\ LE_{4}=0,\\ LE_{5}=0,\\ LE_{6}=0,\\ LE_{7}=-0.011,\\ LE_{8}=-0.024,\\ LE_{9}=-0.094,\\ LE_{10}=-0.597. \end{array}\right. \end{array}$$ (4)

As shown in Fig 2, system (1) has *LE*_1,2_ > 0, *LE*_3,4,5,6_ = 0, *LE*_7,8,9,10_ < 0, which means that it exhibits a hyperchaotic behavior with two positive, four zero and four negative *LE*. The sum of the *LE* is also negative, indicating that our suggested 10-D system (1) is dispersed.

According to chaos theory, Kaplan-Yorke dimensions high value directly corresponds to system dynamics’ high complexity. For the proposed system (1), the analogous Kaplan-Yorke dimension is calculated as follows:

$$\begin{array}{c} D_{KY}=j+\frac{1}{|L_{j+1}|}\sum_{j=1}^{j}L_{j}. \end{array}$$ (5)

with *j* representing the index such that:

$$\begin{array}{c} \sum_{i=1}^{j}L_{j}>0\;and\;\sum_{i=1}^{j+1}L_{j}<0. \end{array}$$ (6)

So, for (1), we discover that:

$$\begin{array}{c} D_{KY}=9+\frac{\sum_{i+1}^{9}LE_{i}}{|LE_{10}|}=9.045. \end{array}$$ (7)

We can observe from (7) that fractal dimension of Kaplan-Yorke is very large in comparison to other systems. Thus, the proposed 10-D system (1) displays a very complex hyperchaotic behaviour.

Table 1 illustrate our new system (1) Kaplan-Yorke fractal dimension and that of some famous high dimensional recently reported hyperchaotic systems.

**Table 1:** Kaplan-Yorke fractal dimension of ten high dimensional chaotic system.

System	Fractal dimension
7-D Varan system [30]	2.175
7-D Lagmiri system [10]	2.091
7-D Yu system [31]	5.278
7-D Yang system [32]	6.149
7-D Hu and Chan system [33]	6.732
9-D Zhu system [12]	2.171
9-D Mahmoud system [13]	5.065
9-D Mahmoud system [14]	5.128
10-D Jianliang system [15]	2.429
The new 10-D system (1)	9.045

In 2011, J. C. Sprott [29] proposed three criteria for the publication of a new hyperchaotic system. It is said in [29], that a new system must satisfy at least one criterion. Among the three criteria, one criterion is that the system should exhibit some behavior previously unobserved. The new behavior of the new 10D hyperchaotic is compared in Table 1.

It can be seen from Table 1 that the 10-D system (1) has a more advanced fractal dimension than some famous high dimensional chaotic systems reported in literature, which indicate and prove the high complexity of system (1).

## Dynamical analysis of the new 10-D hyperchaotic system

In this part, the effect of initial conditions and coefficient on the complexity and properties of system (1) would be studied. Stability of equilibrium points, Lyapunov exponents, fractal dimension and coexisting attractors will be the main properties of investigation.

### Equilibrium points and stability

The first step in dynamic analysis is solving the algebraic equations below to discover new 10-D hyperchaotic system (1) points of equilibrium:

$$\begin{array}{c} \left\{ \begin{array}{c} x_{3}+x_{1}x_{2}-x_{1}=0,\\ 1+a(x_{2}-x_{1}^{4})-x_{1}^{2}=0,\\ -x_{1}+x_{3}+x_{4}=0,\\ -bx_{3}+cx_{5}=0,\\ -x_{4}+x_{6}=0,\\ -x_{5}+x_{7}=0,\\ -x_{6}+x_{8}=0,\\ -x_{7}+(1-d)x_{9}=0,\\ -x_{8}+x_{10}=0,\\ -x_{9}+x_{7}=0. \end{array}\right. \end{array}$$ (8)

By considering the parameters values (3), three equilibrium points is obtained as the following:

$$\begin{array}{c} \left\{ \begin{array}{c} E_{1}=[0,-10,0,0,0,0,0,0,0,0]\\ E_{2}=[-1,1,0,-1,0,-1,0,-1,0,-1]\\ E_{3}=[1,1,0,1,0,1,0,1,0,1] \end{array}\right. \end{array}$$ (9)

We study the stability of (1) at the three equilibrium points by studying the eigenvalues of the following Jacobean of the 10-D system.

$$\begin{array}{c} J_{E_{i}}=[\begin{array}{cccccccccc} x_{2}-1 & x_{1} & 1 & 0 & 0 & 0 & 0 & 0 & 0 & 0\\ -2x_{1}-4ax_{1}^{3} & a & 0 & 0 & 0 & 0 & 0 & 0 & 0 & 0\\ -1 & 0 & 1 & 1 & 0 & 0 & 0 & 0 & 0 & 0\\ 0 & 0 & -b & 0 & c & 0 & 0 & 0 & 0 & 0\\ 0 & 0 & 0 & -1 & 0 & 1 & 0 & 0 & 0 & 0\\ 0 & 0 & 0 & 0 & -1 & 0 & 1 & 0 & 0 & 0\\ 0 & 0 & 0 & 0 & 0 & -1 & 0 & 1 & 0 & 0\\ 0 & 0 & 0 & 0 & 0 & 0 & 1 & 0 & 1-d & 0\\ 0 & 0 & 0 & 0 & 0 & 0 & 0 & -1 & 0 & 1\\ 0 & 0 & 0 & 0 & 0 & 0 & 1 & 0 & -1 & 0 \end{array}] \end{array}$$ (10)

By considering the parameters values (3) the characteristic polynomial of $J_{E_{i}}$ is calculated as:

$$\begin{array}{c} \nabla \left(\lambda \right)=\lambda^{10}+9.9\lambda^{9}-4.81\lambda^{8}+62.381\lambda^{7}-57.222\lambda^{6}+104.3812\lambda^{5}-101.0089\lambda^{4}+36.6971\lambda^{3}-24.8579\lambda^{2}+2.2209\lambda -0.0011. \end{array}$$ (11)

Then, the eigenvalues of $J_{E_{1}}$ are obtained as:

$$\begin{array}{c} \lambda_{1}=-10.3161,\lambda_{2,3}=\pm 1.9358i,\lambda_{4}=0.2538,\lambda_{5,6}=0.0058\pm 1.4453i,\lambda_{7,8}=0.0245\pm 0.5498i,\lambda_{9}=0,\lambda_{10}=0.1. \end{array}$$ (12)

The characteristic polynomial of $J_{E_{2}}$ is calculated as:

$$\begin{array}{c} \nabla \left(\lambda \right)=\lambda^{10}-1.1\lambda^{9}+9.69\lambda^{8}-9.209\lambda^{7}+31.433\lambda^{6}-25.5918\lambda^{5}+36.7331\lambda^{4}-25.9284\lambda^{3}+8.9336\lambda^{2}-5.5251\lambda +0.0024. \end{array}$$ (13)

Then, the eigenvalues of $J_{E_{2}}$ are obtained as:

$$\begin{array}{c} \lambda_{1,2}=0.169\pm 1.8045i,\lambda_{3,4}=0.0021\pm 1.9349i,\lambda_{5,6}=0.0015\pm 1.4339i,\lambda_{7}=0.7185,\lambda_{8,9}=0.0179\pm 0.549i,\lambda_{10}=0. \end{array}$$ (14)

The characteristic polynomial of $J_{E_{3}}$ is calculated as:

$$\begin{array}{c} \nabla \left(\lambda \right)=\lambda^{10}+9.9\lambda^{9}-4.81\lambda^{8}+62.381\lambda^{7}-57.22\lambda^{6}+104.3812\lambda^{5}-101.0089\lambda^{4}+36.697\lambda^{3}-24.8579\lambda^{2}+2.2209\lambda -0.0011. \end{array}$$ (15)

Then, the eigenvalues of $J_{E_{3}}$ are obtained as:

$$\begin{array}{c} \lambda_{1}=-10.9161,\lambda_{2},3=\pm 1.9358i,\lambda_{4}=0.8538,\lambda_{5},6=0.0058\pm 1.4453i,\lambda_{7},8=0.0245\pm 0.5498i,\lambda_{9}=0,\lambda_{1}0=0.1. \end{array}$$ (16)

We observe the existence of four eigenvalues with positive real part in (12), nine positive eigenvalues in (14) and nine positive eigenvalues in (16) which shows that all equilibrium points are unstable. In addition, we can conclude that the 10-D system hyperchaotic attractor belongs to the self-excited family (1).

### Bifurcation, Lyapunov exponents and fractal dimension

The *LE* spectrum and bifurcation diagram are two most significant tools for analyzing a system’s dynamical behavior. The Kaplan-Yorke fractal dimension is also a useful indicator of system complexity. The dynamical behavior and complexity of the novel 10-D system (1) are examined using numerical simulations in this section of the study, with variable positive coefficient *a*, *b*, *c*, and *d*.

#### Parameter *a* varying

**Fig 3:**
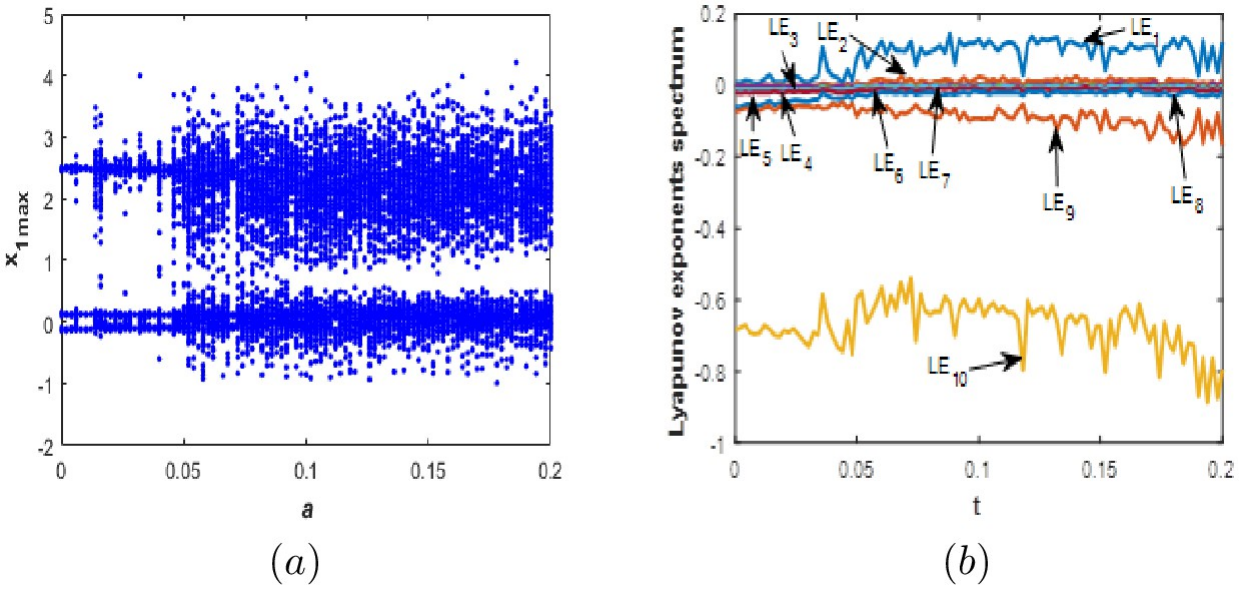
Bifurcation diagram (a) and Lyapunov exponents spectrum (b) of the new 10-D system (1) when: *b* = 0.1, *c* = 1.8, *d* = 0.01 and *a* ∈ [0;0.2].

To investigate the sensitivity of (1) to the value of parameter *a*, we let *b* = 0.1, *c* = 0.5, *d* = 0.01 and vary *a* between 0 and 0.2. The bifurcation diagram (*BD*) of (1) with corresponding Lyapunov exponents spectrum when *a* belongs to the following set of values [0;0.2] and for initial conditions (3) are depicted in Fig 3, we can observe that *BD* and Lyapunov exponents spectrum are in good agreement. Fig 3 illustrate that the new 10-D system (1) can exhibit periodic behavior without positive Lyapunov exponents which means that the Kaplan-yorke fractal dimension equal to zero indicating no complexity of the dynamics. Also, the 10-D system can involves into a chaotic attractor or a hyperchaotic attractor with high Kaplan-Yorke fractal dimension which indicates complexity of the dynamics.

**Fig 4:**
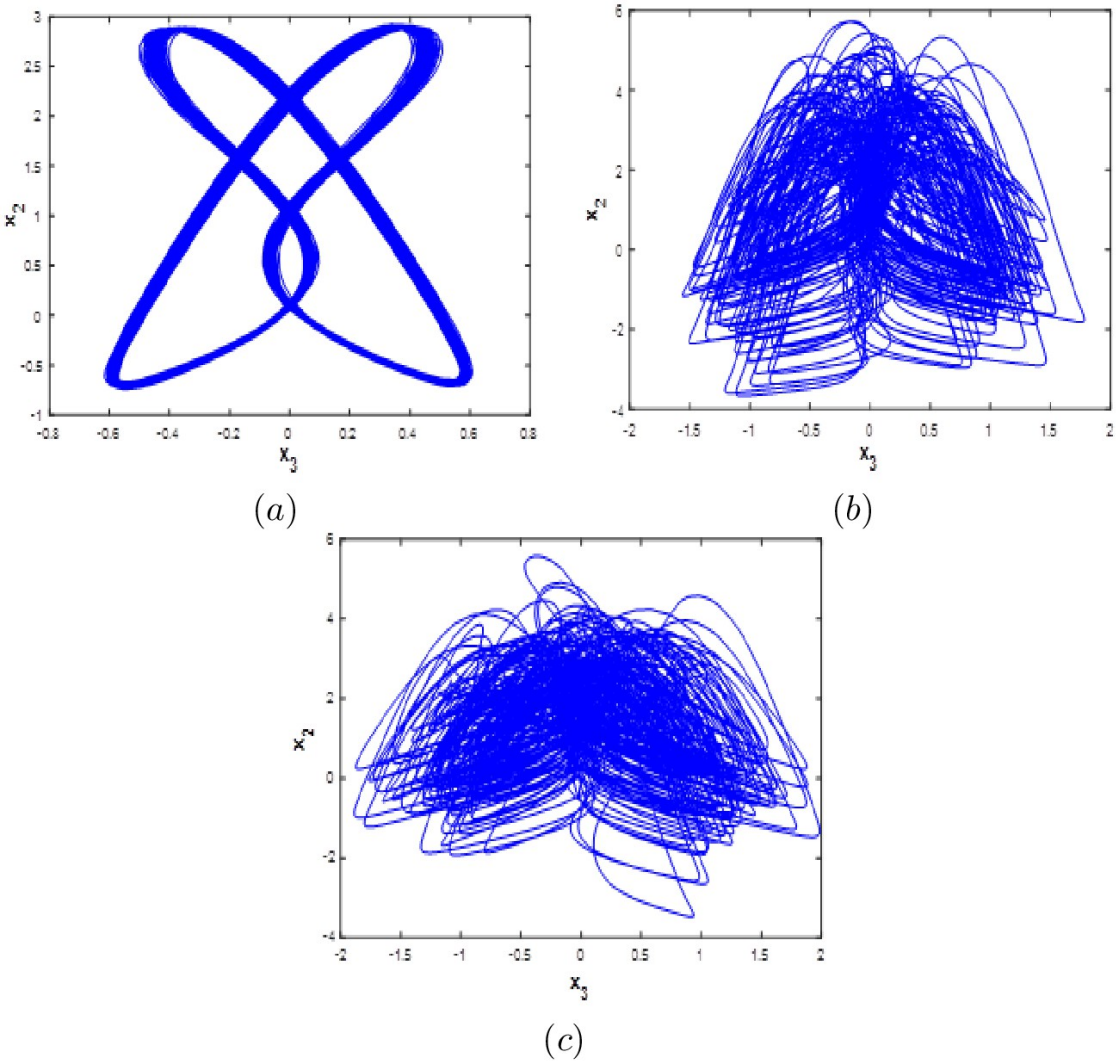
Phase portraits of the new 10-D system (1) for different values of *a*. (a) *x*_3_ − *x*_2_ Quasi-periodic attractor, (b) *x*_3_ − *x*_2_ chaotic attractor and (c) *x*_3_ − *x*_2_ hyperchaotic attractor.

When *a* ∈ ([0, 0.013], [0.017, 0.021], [0.027, 0.031]), the new 10-D system (1) exhibit a periodic behavior without complexity. When *a* ∈ ([0.014, 0.016], [0.022, 0.026], [0.032, 0.042], [0.046, 0.050], [0.188, 2]), the new 10-D system (1) generates a chaotic behaviour with different level of complexity. When *a* varies the value of kaplan-yorke dimension moves from 6.739 when *a* = 0.035 to 8.374 when *a* = 0.195. When *a* ∈ ([0.050, 0.187]), the new 10-D system (1) exhibits a hyperchaotic behavior with very high level of complexity. The corresponding kaplan-yorke dimension moves from a high value of 8.746 when *a* = 0.185 to a very high value of 9.067 when *a* = 0.6. Fig 4 illustrates several attractors and dynamical behaviors for various values of *a*. In addition, Table 2 shows the Kaplan-Yorke fractal dimension and Lyapunov exponents for some values of *a*.

**Table 2:** Lyapunov exponents, Kaplan-Yorke dimensin and dynamics of the new 10D system (1) with parameter *a* varying.

*a*	*LE*_1_	*LE*_2_	*LE*_3_	*LE*_4_	*LE*_5_	*LE*_6_	*LE*_7_	*LE*_8_	*LE*_9_	*LE*_10_	*D*_*KY*_	Dynamics
0.06	0.132	0.015	0	0	0	0	-0.011	-0.018	-0.079	-0.578	9.067	Hyperhaos
0.185	0.111	0.014	0	0	0	0	-0.016	-0.015	-0.126	-0.723	8.746	Hyperhaos
0.195	0.134	0	0	0	0	-0.01	-0.012	-0.023	-0.238	-0.711	8.374	Chaos
0.035	0.027	0	0	0	0	-0.01	-0.023	-0.038	-0.056	-0.69	6.739	Chaos
0.01	0	0	0	0	-0.011	-0.012	-0.02	-0.056	-0.07	-0.693	0	Quasi-Periodic

#### Parameter *b* varying

**Fig 5:**
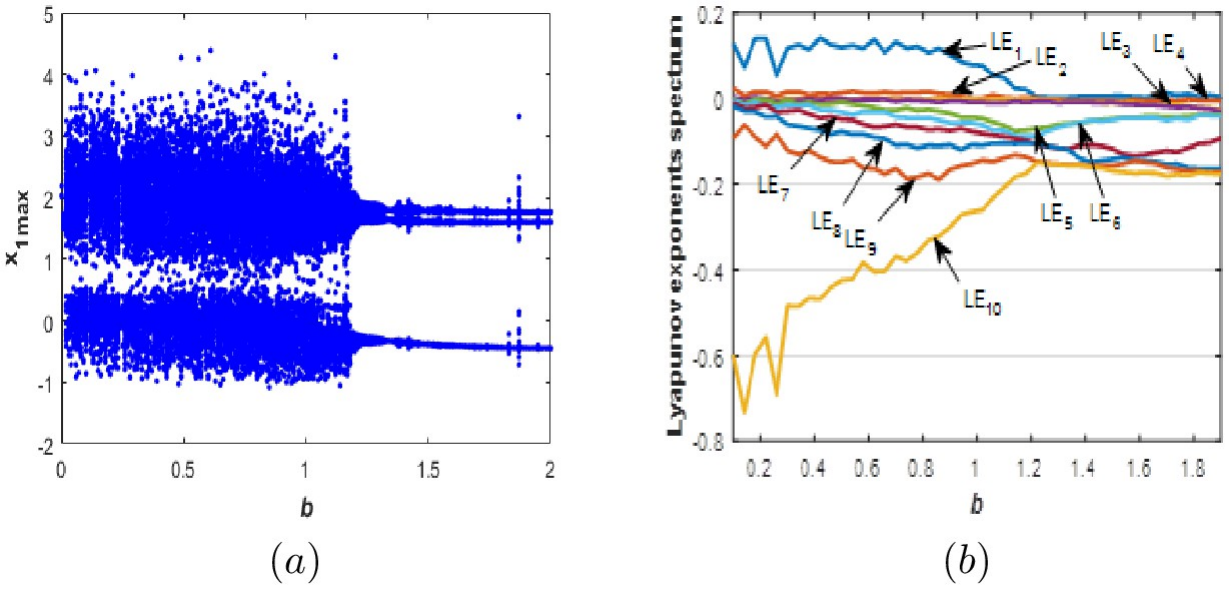
Bifurcation diagram (a) and Lyapunov exponents spectrum (b) of the new 10-D system (1) when: *a* = 0.1, *c* = 1.8, *d* = 0.01 and *b* ∈ [0.1;2].

To investigate the sensitivity of new 10-D hyperchaotic system (1) to the value of parameter *b*, we fix *a* = 0.1, *c* = 1.8, *d* = 0.01 and vary *b* between 0.1 and 2. Fig 5 gives the *LE* spectrum and *BD* of (1) when and for initial conditions (3), we can observe an excellent compatibility between *LE* spectrum and the corresponding *BD*.

It is obvious from Fig 5 that the proposed 10-D system (1) can exhibits periodic behaviour with Kaplan-yorke fractal dimension equal to zero indicating no complexity of the dynamics. Also, the 10-D system can involves into a chaotic attractor with one positive *LE* and a higher fractional Kaplan-Yorke dimension which indicates complexity of the dynamics. In addition, more complexity is observed when the new system generate a hyperchaotic behaviour with more than one positive *LE* and higher values of Kaplan-Yorke fractal dimension, which indicates a very complicated dynamic behavior generated by the new 10-D system (1).

**Fig 6:**
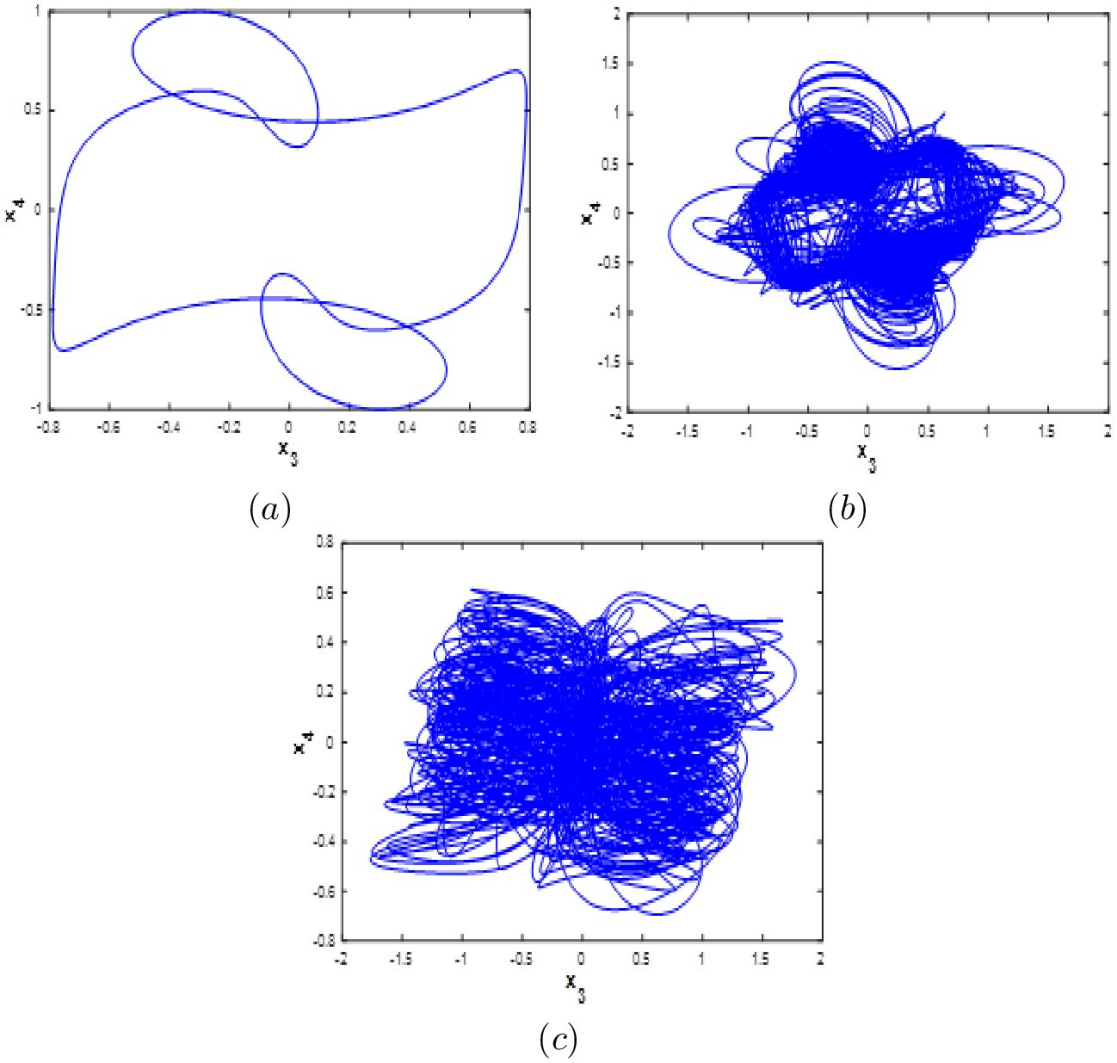
Phase portraits of the new 10-D system (1) for different values of *b*. (a)*x*_3_ − *x*_4_Periodic attractor, (b) *x*_3_ − *x*_4_ chaotic attractor and (c) *x*_3_ − *x*_4_ hyperchaotic attractor.

When, the new 10-D system (1) associate into a hyperchaotic attractor with two positive *LE* and a very high complexity. The corresponding Kaplan-Yorke fractal dimension equal to: 6.597 when *b* = 0.8, 7.960 when *b* = 0.42, 8.165 when *b* = 0.3 and it can reach 9.064 when *b* = 0.1. These high values prove the very complex behavior of system (1). When, the new 10-D system (1) with one positive Lyapunov exponents generates a chaotic behavior, the corresponding Kaplan-Yorke fractal dimension equal to: 5.236 when *b* = 0.95 and 4.465 when *b* = 1.16. When, the new 10-D system (1) possesses a quasi-periodic behavior with two or three zero and eight negative *LE*s. Different dynamic behaviors and attractors for special values of the parameter*b* are displayed in Fig 6. Table 3 shows the *LE* and the Kaplan-Yorke fractal dimension for different values of *b*.

**Table 3:** Lyapunov exponents, Kaplan-Yorke dimension and dynamics of the new 10D system (1) with parameter *b* varying.

*b*	*LE*_1_	*LE*_2_	*LE*_3_	*LE*_4_	*LE*_5_	*LE*_6_	*LE*_7_	*LE*_8_	*LE*_9_	*LE*_10_	*D*_*KY*_	Dynamics
0.1	0.135	0.023	0	0	0	-0.01	-0.011	-0.014	-0.085	-0.593	9.064	Hyperchaos
0.3	0.12	0.014	0	0	-0.01	-0.013	-0.03	-0.061	-0.121	-0.497	8.165	Hyperchaos
0.42	0.129	0.016	0	0	-0.016	-0.022	-0.035	-0.075	-0.134	-0.468	7.96	Hyperchaos
0.8	0.118	0.013	0	-0.01	-0.029	-0.055	-0.062	-0.113	-0.176	-0.377	6.597	Hyperhaos
0.95	0.066	0	0	-0.01	-0.043	-0.055	-0.069	-0.107	-0.156	-0.253	5.236	Chaos
1.16	0.046	0	0	-0.013	-0.071	-0.088	-0.095	-0.109	-0.139	-0.188	4.465	Chaos
1.5	0	0	-0.01	-0.01	-0.047	-0.048	-0.119	-0.143	-0.157	-0.167	0	Quasi-Periodic

#### Parameter *c* varying

**Fig 7:**
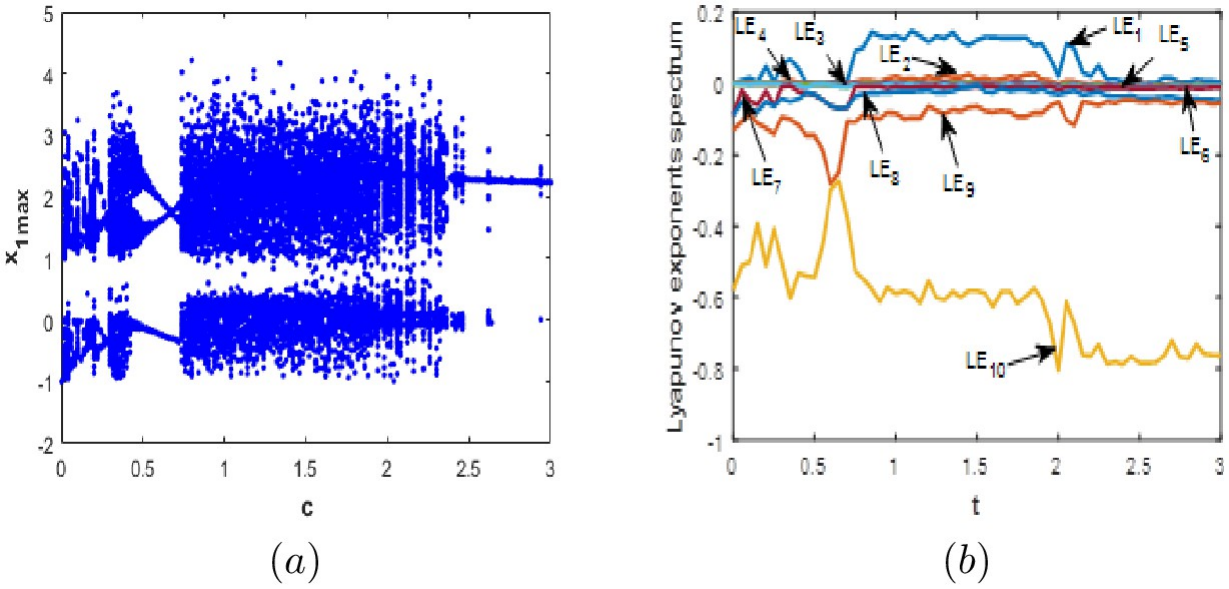
Bifurcation diagram (a) and Lyapunov exponents spectrum (b) of the new 10-D system (1) when: *a* = 0.1, *b* = 0.1, *d* = 0.01 and *c* ∈ [0; 3].

**Fig 8:**
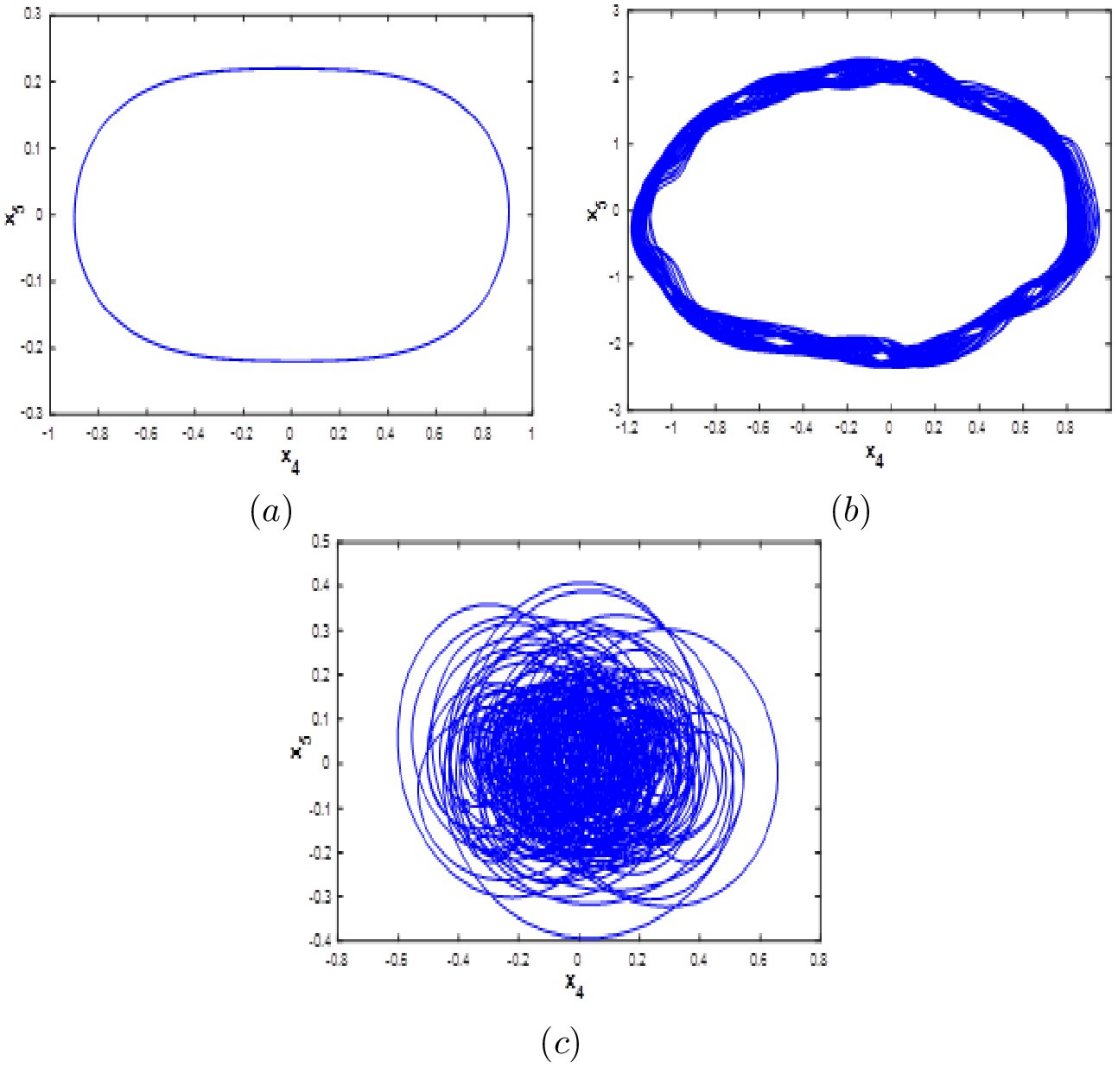
Phase portraits of the new 10-D system (1) for different values of *c*. (a) *x*_4_ − *x*_5_ Periodic attractor, (b) (a) *x*_4_ − *x*_5_ chaotic attractor and (c) (a) *x*_4_ − *x*_5_ hyperchaotic attractor.

To study 10-D system (1) sensitivity to the value of parameter *c*, we let *a* = 0.1, *b* = 0.1, *d* = 0.01 and vary *c* between 0 and 3. Fig 7 gives the *LE* spectrum and *BD* of system (1) when and for initial conditions (3). From close observation of the figure, there is a good compatibility between Lyapunov exponents spectrum and the corresponding bifurcation diagram. When parameter *c* varies, we can see from Fig 7 that the new 10-D system (1) can exhibits periodic behavior with no complexity, chaotic behavior with one positive *LE* and a high fractional Kaplan-Yorke dimension, which illustrates complexity of the dynamics. In addition, higher complexity is observed when the proposed system generates a hyperchaotic behavior with more than one positive *LE* and higher values of fractional Kaplan-Yorke dimension. When *c* ([0, 0.05], [0.13, 0.15], [0.24, 0.26], [0.45, 0.70], [2.36, 3]) the new system (1) generates periodic behaviour where the corresponding Kaplan-Yorke fractal dimension equal to zero. When *c* ([0.06, 0.012], [0.17, 0.23], [0.27, 0.44], [0.71, 0.8], [1.92, 2.35], [2.65, 2.70], [2.85, 2.87]) the new 10-D system (1) involves into a chaotic attractor with different level of complexity. When *c* = 0.1, the value of Kaplan-Yorke fractal dimension is 6.676. This value may increase up to 7.163 when *c* = 0.40 providing a high complexity. When *c* = 0.77 we have obtained the highest value for chaotic attractors of system (1) which is 8.589. When, the new 10-D system (1) displays a hyperchaotic behavior with two positive, four zero and four negative *LE*. The corresponding Kaplan-Yorke fractal dimension is 9.031 when *c* = 1.4. Different attractors and dynamical behaviors for special values of the coefficient *c* are given in Fig 8. Table 4 shows the *LE* and fractional Kaplan-Yorke dimension for various values of *c*.

**Table 4:** Lyapunov exponents, Kaplan-Yorke dimension and dynamics of the new 10D system (1) with parameter *c* varying.

*c*	*LE*_1_	*LE*_2_	*LE*_3_	*LE*_4_	*LE*_5_	*LE*_6_	*LE*_7_	*LE*_8_	*LE*_9_	*LE*_10_	*D*_*KY*_	Dynamics
1.4	0.121	0.018	0	0	0	0	-0.010	-0.019	-0.091	-0.608	9.031	Hyperchaos
0.77	0.113	0	0	0	0	0	-0.010	-0.040	-0.107	-0.533	8.589	Chaos
0.4	0.041	0	0	0	0	-0.010	-0.024	-0.043	-0.117	-0.531	7.163	Chaos
0.1	0.025	0	0	0	0	0	-0.037	-0.072	-0.092	-0.502	6.676	Chaos
2.6	0	0	-0.010	-0.010	-0.011	-0.012	-0.014	-0.035	-0.049	-0.773	0	Quasi-Periodic

#### Parameter *d* varying

To examine the 10-D system (1) sensitivity for the value of coefficient *d*, we let *a* = 0.1, *b* = 0.1, *c* = 1.8 and vary *d* between 0 and 1. Fig 8 gives the *LE* spectrum and *BD* of system (1) when and for initial conditions (3), it is obvious to notice the good compatibility between Lyapunov exponents spectrum and the corresponding bifurcation diagram.

**Fig 9:**
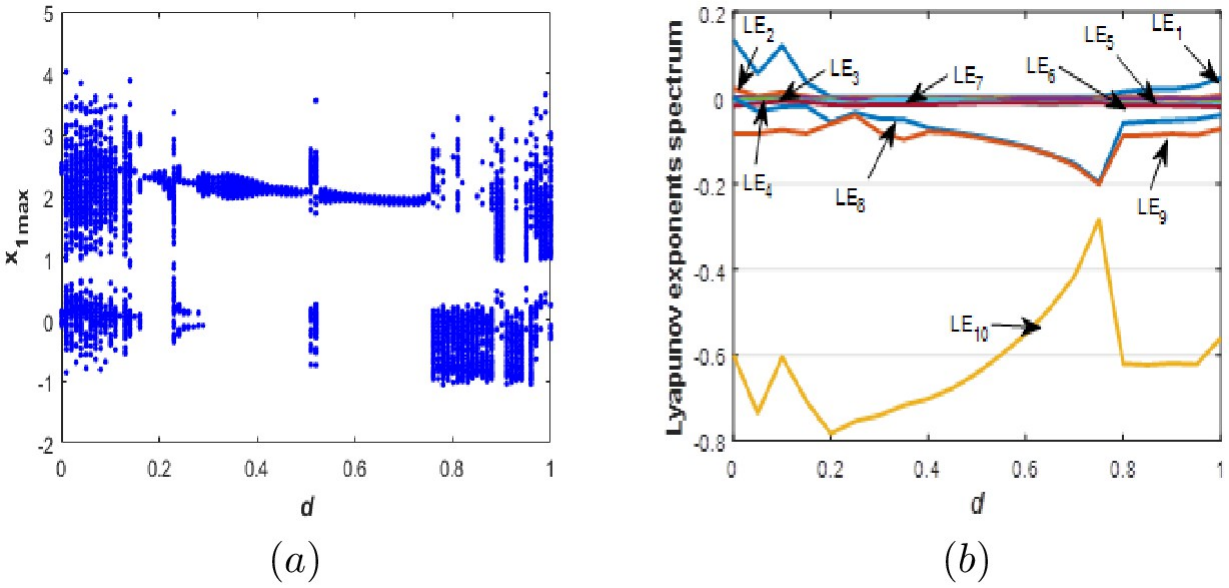
Bifurcation diagram (a) and Lyapunov exponents spectrum (b) of the new 10-D system (1) when: *a* = 0.1, *b* = 0.1, *c* = 1.8 and *d* ∈ [0; 1].

**Fig 10:**
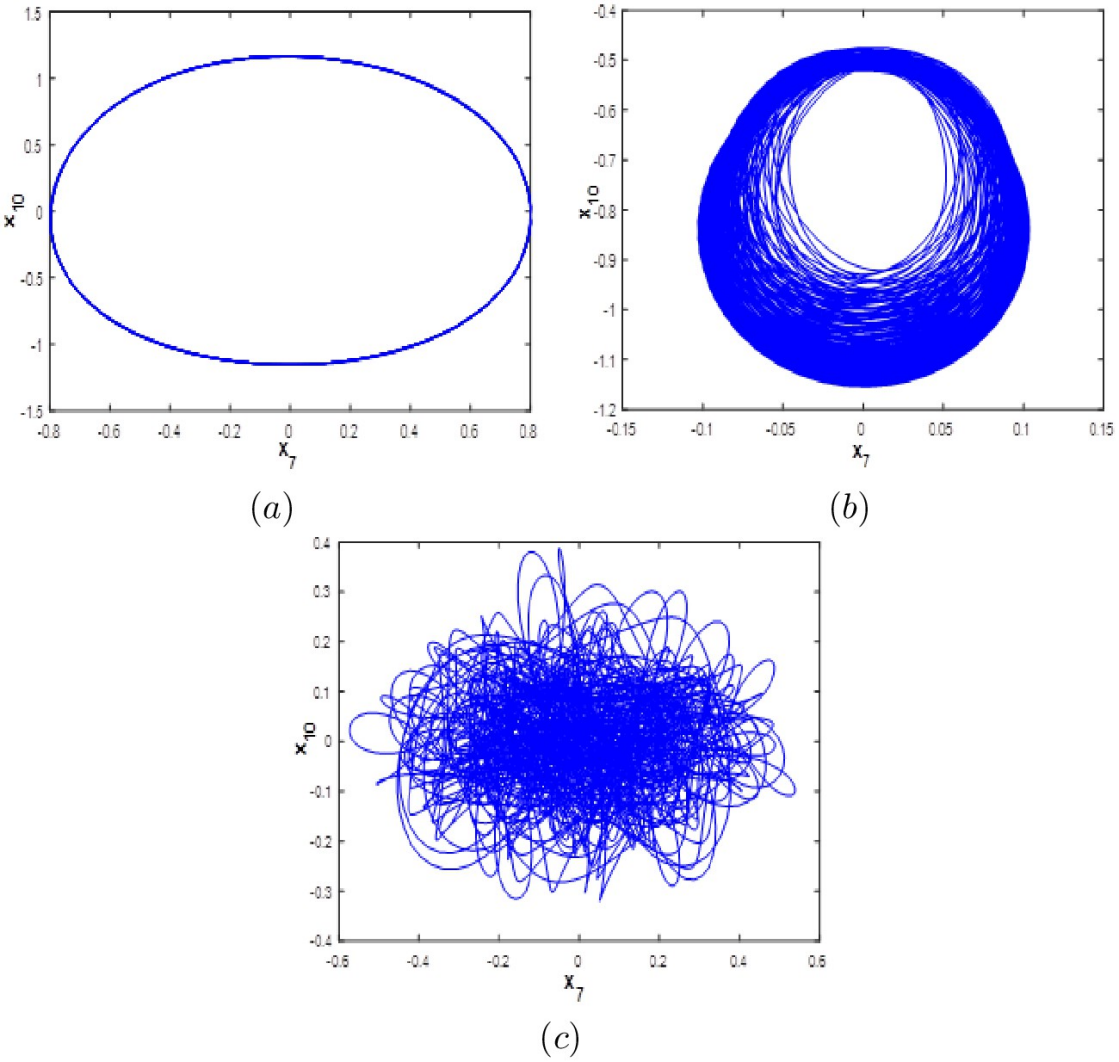
Phase portraits of the new 10-D system (1) for various values of *d*. (a) *x*_7_ − *x*_10_ Periodic attractor, (b) *x*_7_ − *x*_10_ chaotic attractor and (c) *x*_7_ − *x*_10_ hyperchaotic attractor.

When parameter *d* varies, we can see from Fig 9 that the new 10-D system (1) can exhibits periodic behavior with no complexity, chaotic behavior with one positive *LE* and a high fractional Kaplan-Yorke dimension, which implies complexity of the dynamics. In addition, higher complexity is observed when (1) generates a hyperchaotic behavior with more than one positive *LE* and higher values of Kaplan-Yorke fractal dimension. When the new system (1) generates Hyperchaotic behaviour with very high complexity where the corresponding Kaplan-Yorke fractal dimension is about 9.028 when *d* = 0.02. When the new 10-D system (1) involves into a chaotic attractor where the Kaplan-Yorke fractal dimension is about 4.667 when *d* = 0.16, 5.33 when *d* = 0.85 and may increase up to 7.937 when *d* = 0.05 indicating more complexity. When the new 10-D system (1) exhibits a quasi-periodic behavior. Various types of attractors and dynamic behaviors for special values of the parameter *d* are presented in Fig 10. Table 5 shows the *LE*, the Kaplan-Yorke fractal dimension and the dynamics for different values of *d*. To the best of the authors knowledge, this study on the new 10-D hyperchaotic system with a Kaplan-Yorke fractal dimension higher than 9 has never been studied by any researcher.

**Table 5:** Lyapunov exponents, Kaplan-Yorke dimension and dynamics of the new 10-D hyperchaotic system (1) with parameter *d* varying.

*d*	*LE*_1_	*LE*_2_	*LE*_3_	*LE*_4_	*LE*_5_	*LE*_6_	*LE*_7_	*LE*_8_	*LE*_9_	*LE*_10_	*D*_*KY*_	Dynamics
0.02	0.111	0.019	0	0	0	0	-0.010	-0.025	-0.078	-0.599	9.028	Hyperhaos
0.05	0.056	0	0	0	0	-0.012	-0.014	-0.032	-0.083	-0.736	7.937	Chaos
0.85	0.019	0	0	0	-0.014	-0.015	-0.018	-0.055	-0.088	-0.624	5.33	Chaos
0.16	0.010	0	0	0	-0.015	-0.022	-0.023	-0.028	-0.050	-0.786	4.667	Chaos
0.5	0	0	0	0	-0.010	-0.010	-0.013	-0.089	-0.092	-0.642	0	Quasi-Periodic

### Multistability and coexisting attractors in the new 10D hyperchaotic system

To study the effect of initial criteria on the behaviour of (1), the bifurcation diagrams of (1) versus its three parameters (*a*, *b* and *c*) for six different initial conditions are calculated and plotted. The obtained bifurcation diagrams allow us to examine the phenomena of multistability; this strange occurrence demonstrates system (1)’s extraordinary sensitivity to initial conditions, which is attributable to its extremely complicated dynamics [34].

Let *ξ*_1_, *ξ*_2_, *ξ*_3_, *ξ*_4_, *ξ*_5_ and *ξ*_6_ be six different initial conditions for the new 10-D hyperchaotic system (1), where:

*ξ*_1_ = (1, 0, 0, 0, 0, 0, 0, 0, 0, 0) (*Bluecolour*)
*ξ*_2_ = (0, 0, 0, 0, 0, 0, 0, 0, 0, 1) (*Redcolour*)
*ξ*_3_ = (0, 0, 0, 0, 0, 0, 0, 0, 0, −1) (*Greencolour*)
*ξ*_4_ = (0, 0, 0, 0, 0, 0, 0, 0, 0, 0.5) (*Magentacolour*)
*ξ*_5_ = (0, 0, 0, 0, 0, 0, 0, 2, 0, 0) (*Yellowcolour*)
*ξ*_4_ = (0, 0, 0, 0, 0, 0, 0, −2, 0, 0) (*Cyancolour*)

#### Multistability when parameter *a* varying

**Fig 11:**
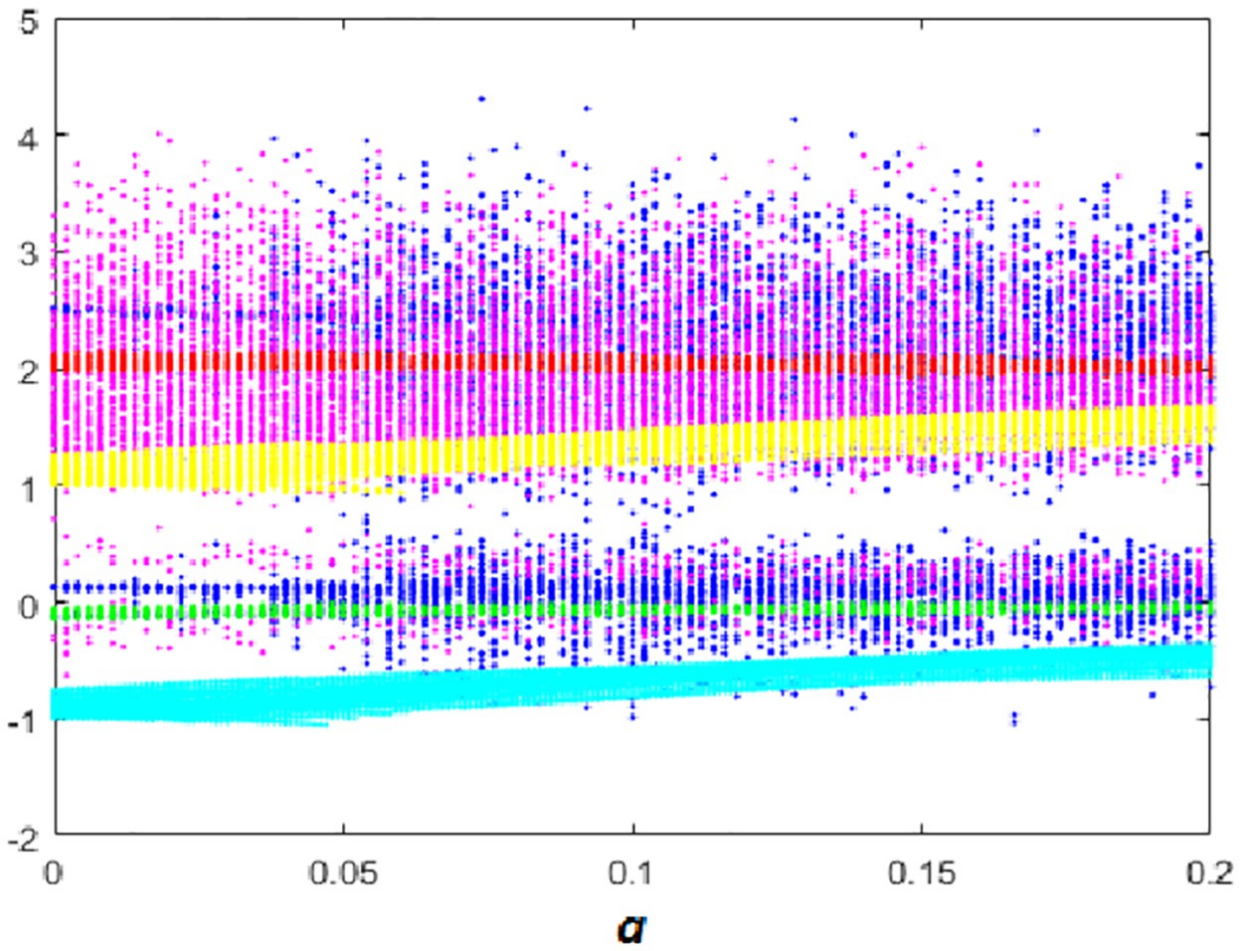
Bifurcation diagram of system (1) versus a starting from:*ξ*_1_ (blue), *ξ*_2_(red), *ξ*_3_(green), *ξ*_4_(magenta), *ξ*_5_(yellow) and *ξ*_6_(cyan).

Here the *BD* of (1) with respect to coefficient *a* is calculated and plotted starting from six different initial points *ξ*_1_, *ξ*_2_, *ξ*_3_, *ξ*_4_, *ξ*_5_ and *ξ*_6_. Fix *b* = 0.1, *c* = 1.8 and *d* = 0.01, from the bifurcation diagram, it can be observed that the new 10D system (1) has six different dynamical evolutions when *a* ∈ [0;0.2] as depicted in Fig 11.

**Fig 12:**
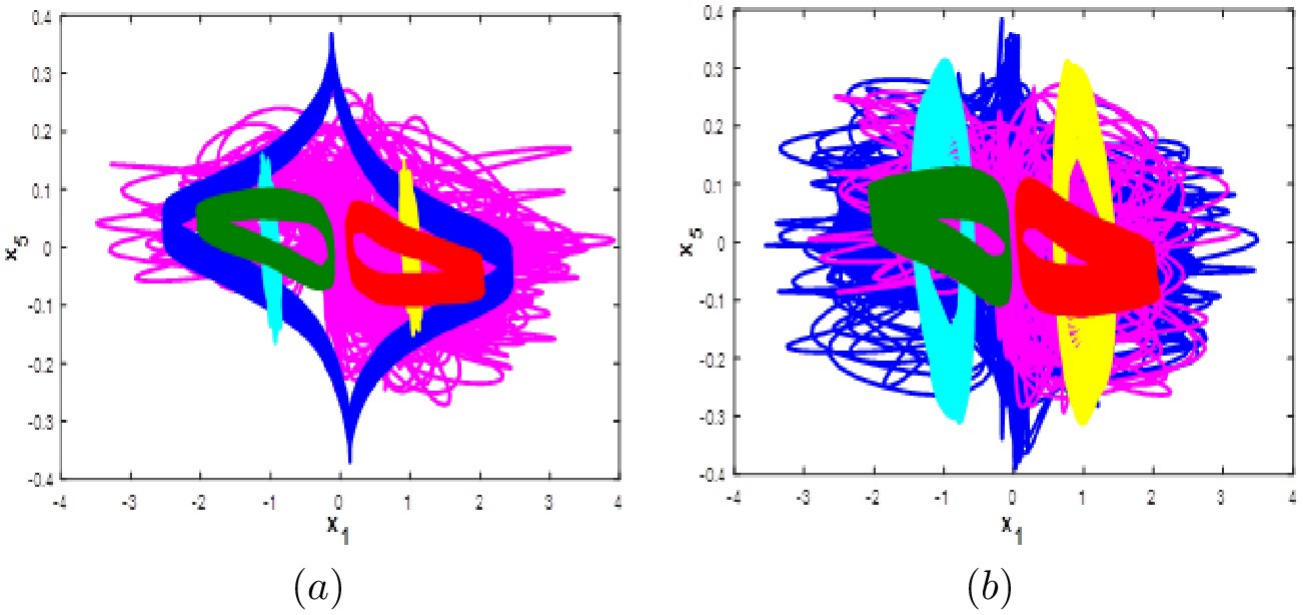
Coexistence of six different attractors projected on the *x*_1_ − *x*_5_ plane. (a) Coexistence of one hyperchaotic attractor and five quasi-periodic attractors when *a* = 0.01. (b) Coexistence of two hyperchaotic attractors and four quasi-periodic attractors when *a* = 0.1.

When *a* ∈ [0;0.04], we can see that system (1) has coexistence of one chaotic attractor starting from and five quasi-periodic attractors as shown in Fig 12(a). Coexistence of four quasi-periodic attractors starting from *ξ*_2_, *ξ*_3_, *ξ*_4_ and *ξ*_3_. and two chaotic attractors starting from *ξ*_1_ and *ξ*_4_ are determined when *a* ∈ [0.05;0.02] as depicted in Fig 12(b). Dynamics, Kaplan-Yorke fractal dimension and Lyapunov exponents for all coexisting attractors when *a* ∈ [0;0.2] are listed in Table 6.

**Table 6:** Lyapunov exponents, Kaplan-Yorke dimensin and dynamics of system (1) coexisisting attractors with parameter *a* varying.

*a*	*ξ*_*i*_	*LE*_1_	*LE*_2_	*LE*_3_	*LE*_4_	*LE*_5_	*LE*_6_	*LE*_7_	*LE*_8_	*LE*_9_	*LE*_10_	*D*_*KY*_	Dynamics
0.01	*ξ*_1_	0	0	0	0	-0.010	-0.012	-0.020	-0.056	-0.070	-0.693	0	Quasi-Periodic
	*ξ*_2_, *ξ*_3_	0	0	0	0	0	-0.010	-0.014	-0.015	-0.305	-0.355	0	Quasi-Periodic
	*ξ*_4_	0.081	0.010	0	0	0	-0.010	-0.011	-0.021	-0.042	-0.527	9.013	Hyperchaos
	*ξ*_5_, *ξ*_6_	0	0	0	0	0	-0.010	-0.013	-0.138	-0.148	-0.658	0	Quasi-Periodic
0.1	*ξ*_1_	0.129	0.024	0	0	0	0	-0.010	-0.018	0.093	-0.597	9.054	Hyperhaos
	*ξ*_2_, *ξ*_3_	0	0	0	0	-0.010	-0.012	-0.024	-0.026	-0.268	-0.403	0	Quais-Periodic
	*ξ*_4_	0.089	0.014	0	0	0	0	-0.010	-0.022	-0.081	-0.533	8.876	Hyperhaos
	*ξ*_5_, *ξ*_6_	0	0	0	-0.010	-0.011	-0.012	-0.015	-0.143	-0.291	-0.476	0	Quais-Periodic

#### Multistability when parameter *b* varying

**Fig 13:**
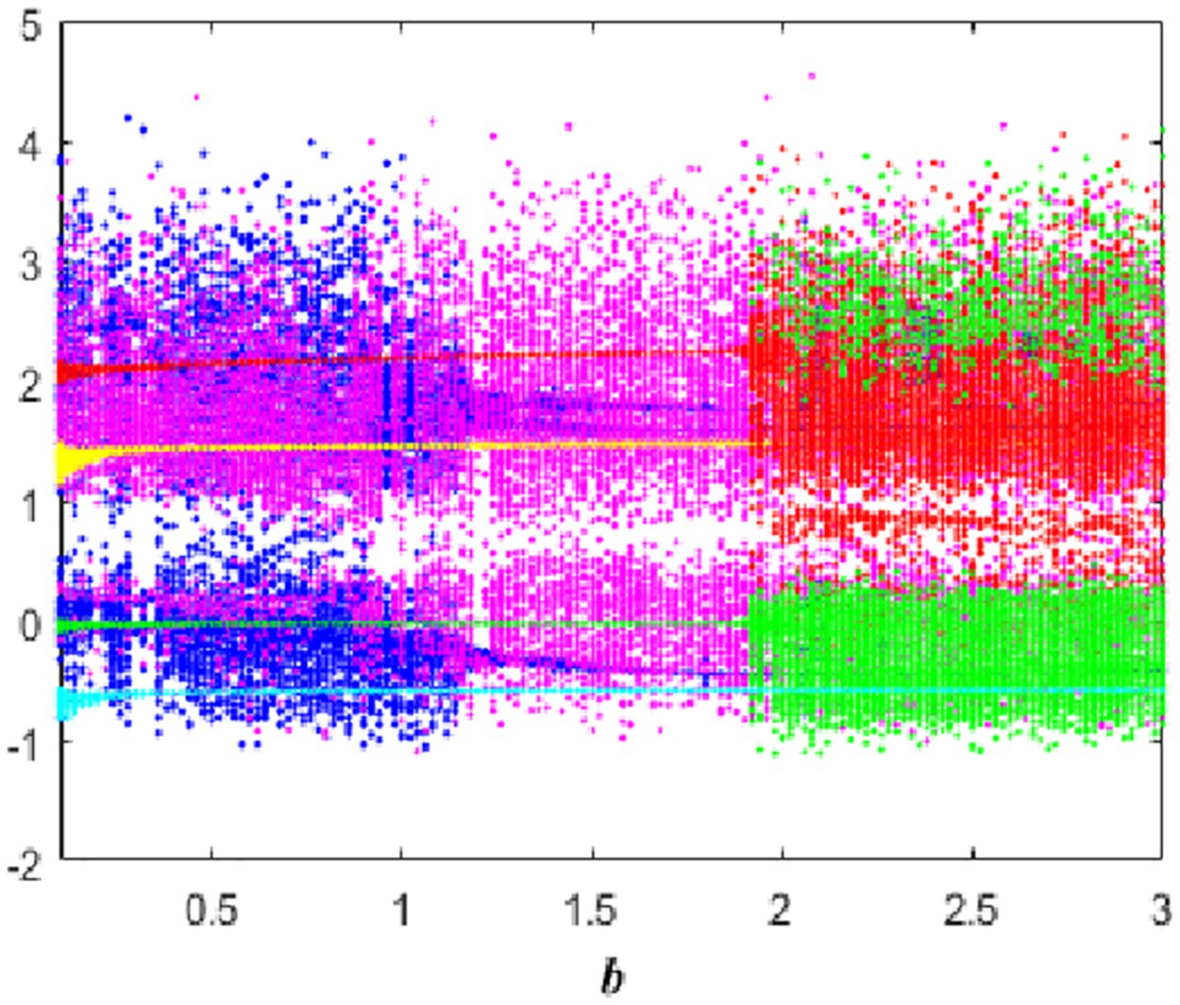
Bifurcation diagram of system (1) versus *b* starting from:*ξ*_1_ (blue), *ξ*_2_(red), *ξ*_3_(green), *ξ*_4_(magenta), *ξ*_5_(yellow) and *ξ*_6_(cyan).

**Fig 14:**
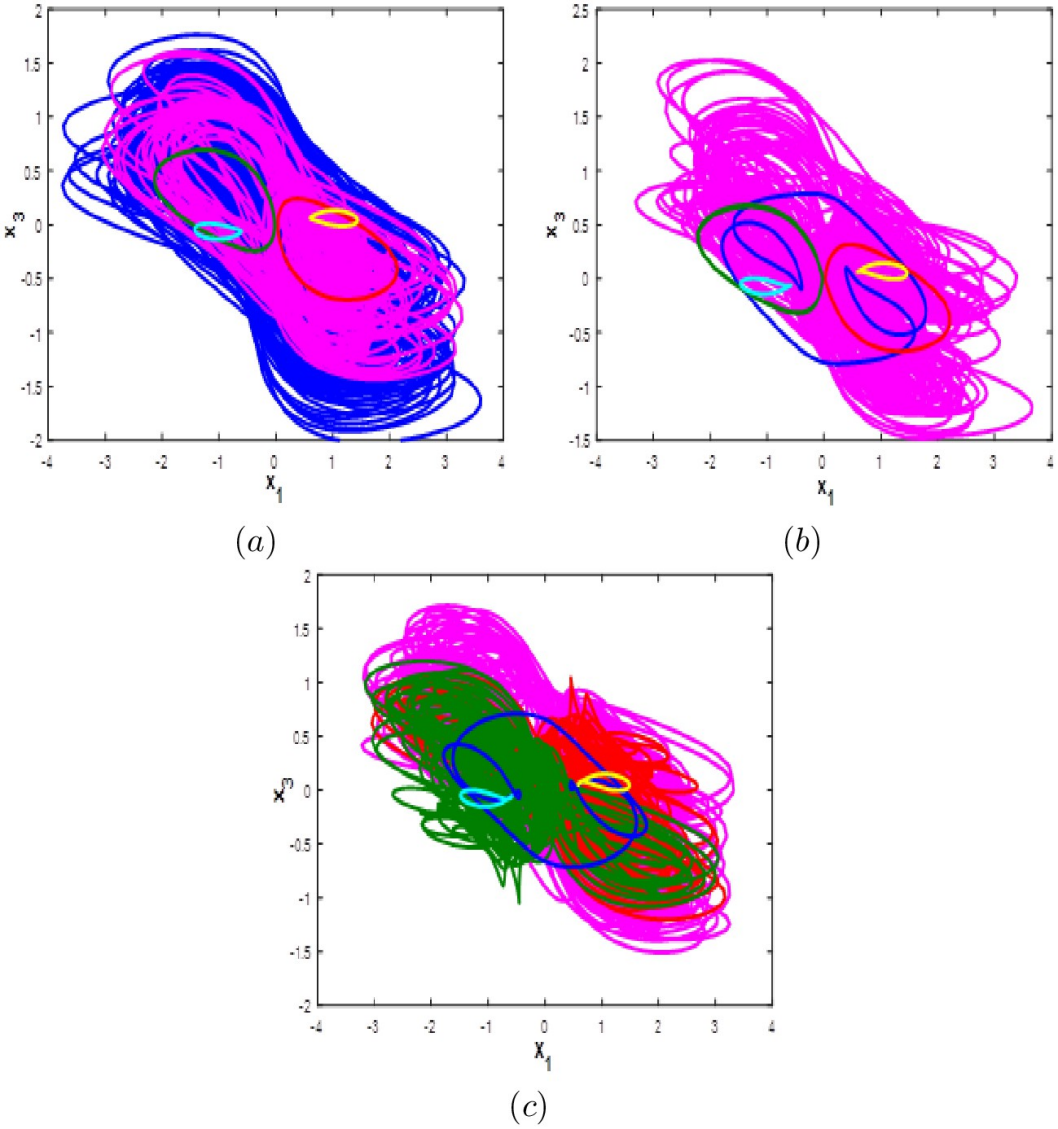
Coexistence of six different attractors projected on the *x*_1_ − *x*_3_ plane. (a) Coexistence of one hyperchaotic attractor (blue), one chaotic attractor (magenta) and four periodic attractors when *b* = 0.5. (b) Coexistence of one chaotic attractor and five periodic attractors when *b* = 1.5. (c) Coexistence of three chaotic attractors and three periodic attractors when *b* = 2.5.

Here system (1) bifurcation diagram with respect to b is calculated and plotted starting from the six different initial points *ξ*_1_, *ξ*_2_, *ξ*_3_, *ξ*_4_, *ξ*_5_ and *ξ*_6_. Fix *a* = 0.1, *c* = 1.8 and *d* = 0.01, from the bifurcation diagram, it can be observed that the new 10D hyperhaotic system (1) exhibit six different dynamical evolutions when *b* ∈ [0.1;3] as depicted in Fig 13. When *b* ∈ [0;1.2], we can see that system (1) has coexistence of two chaotic attractors starting from *ξ*_1_ and *ξ*_4_ and four periodic attractors starting from the remaining initial points as shown in Fig 14(a). Coexistence of one chaotic attractor starting from *ξ*_4_ and five periodic attractor starting from the other initial conditions are observed when *a* ∈ [0.13;0.19], (see Fig 14(b)). When *b* ∈ [2;3], the new 10-D hyperchaotic system has coexistence of three chaotic attractors starting from *ξ*_2_, *ξ*_3_ and *ξ*_4_ and three periodic attractors starting from *ξ*_1_, *ξ*_5_ and *ξ*_6_ as shown in Fig 14(c). Dynamics, Kaplan-Yorke fractal dimension and Lyapunov exponents for all coexisting attractors when *b* ∈ [0.1;3] are listed in Table 7.

**Table 7:** Lyapunov exponents, Kaplan-Yorke dimensin and dynamics of system (1) coexisisting attractors with parameter *b* varying.

*b*	*ξ*_*i*_	*LE*_1_	*LE*_2_	*LE*_3_	*LE*_4_	*LE*_5_	*LE*_6_	*LE*_7_	*LE*_8_	*LE*_9_	*LE*_10_	*D*_*KY*_	Dynamics
0.5	*ξ*_1_	0.119	0.013	0	0	-0.010	-0.032	-0.048	-0.077	-0.149	-0.424	7.545	Hyperhaos
	*ξ*_2_, *ξ*_3_	0	-0.010	-0.015	-0.017	-0.029	-0.032	-0.058	-0.156	-0.192	-0.325	0	Periodic
	*ξ*_4_	0.054	0	0	-0.010	-0.024	-0.032	-0.042	-0.059	-0.121	-0.466	5.625	Chaos
	*ξ*_5_, *ξ*_6_	0	-0.010	-0.027	-0.030	-0.062	-0.064	-0.071	-0.103	-0.289	-0.308	0	Periodic
1.5	*ξ*_1_	0	-0.004	-0.009	-0.010	-0.046	-0.049	-0.119	-0.144	-0.157	-0.167	0	Periodic
	*ξ*_2_, *ξ*_3_	0	-0.010	-0.065	-0.068	-0.076	-0.085	-0.098	-0.100	-0.194	-0.199	0	Periodic
	*ξ*_4_	0.018	0	-0.010	-0.016	-0.051	-0.053	-0.118	-0.139	-0.156	-0.177	3.5	Chaos
	*ξ*_5_, *ξ*_6_	0	-0.010	-0.098	-0.099	-0.112	-0.113	-0.114	-0.133	-0.142	-0.145	0	Periodic
2.5	*ξ*_1_	0	-0.010	-0.028	-0.031	-0.040	-0.047	-0.052	-0.140	-0.213	-0.215	0	Periodic
	*ξ*_2_, *ξ*_3_	0.012	0	-0.030	-0.032	-0.060	-0.061	-0.137	-0.147	-0.216	-0.220	2.4	Chaos
	*ξ*_4_	0.017	0	-0.025	-0.027	-0.038	-0.049	-0.066	-0.145	-0.214	-0.221	2.68	Chaos
	*ξ*_5_, *ξ*_6_	0	-0.010	-0.045	-0.047	-0.110	-0.111	-0.113	-0.115	-0.209	-0.211	0	Periodic

#### Multistability when parameter *c* varying

**Fig 15:**
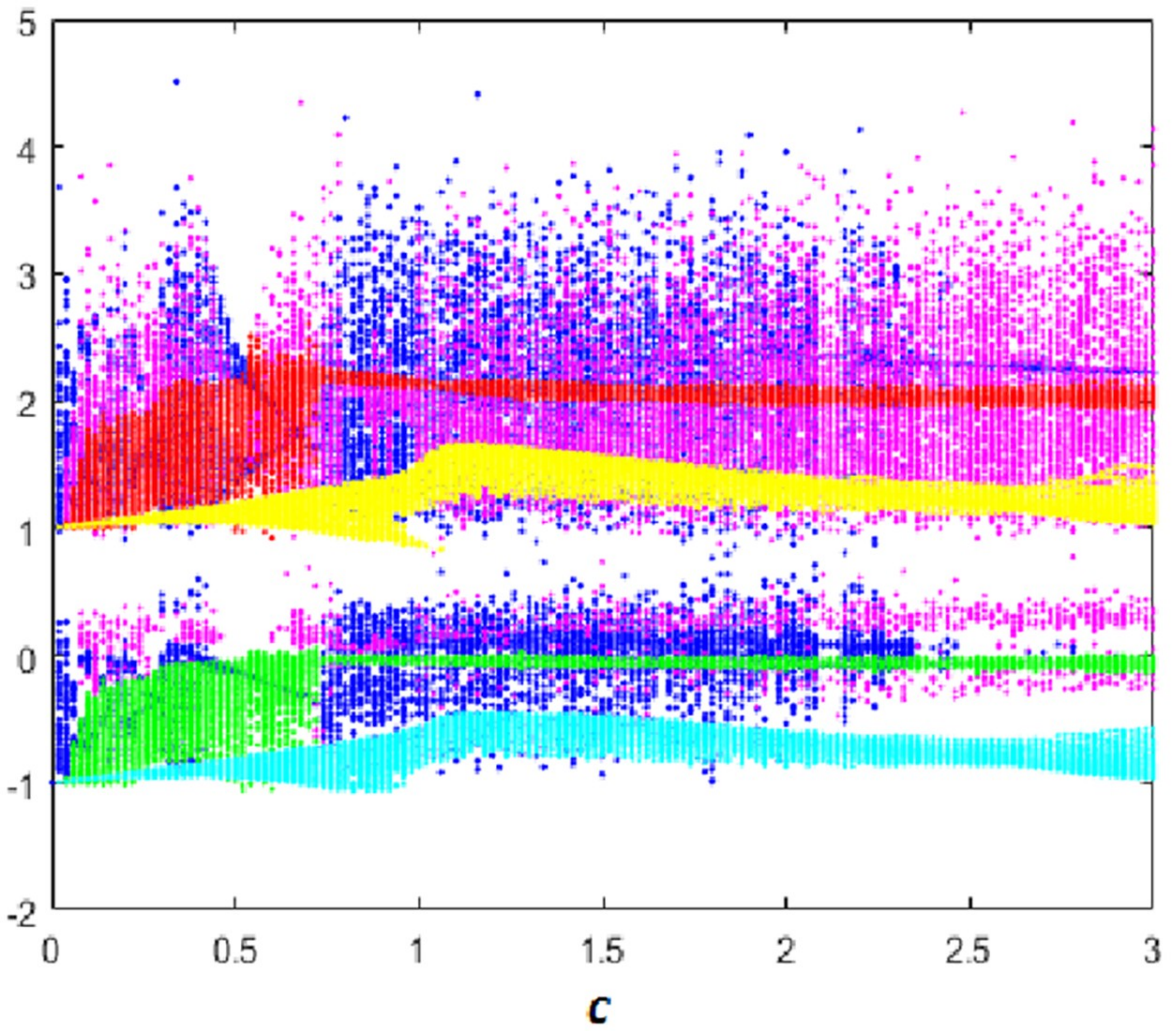
Bifurcation diagram of system (1) versus *c* starting from:*ξ*_1_ (blue), *ξ*_2_(red), *ξ*_3_(green), *ξ*_4_(magenta), *ξ*_5_(yellow) and *ξ*_6_(cyan).

**Fig 16:**
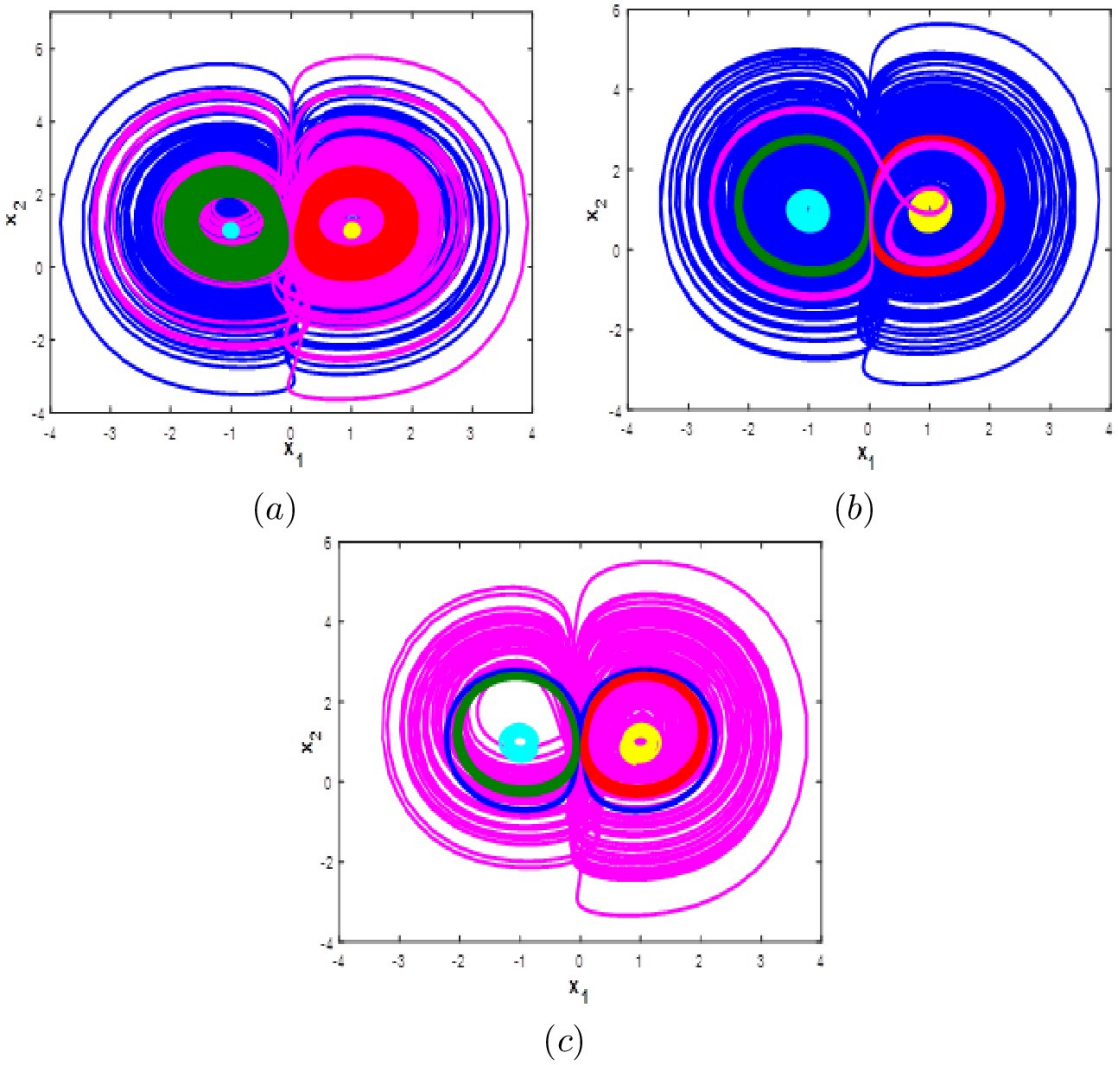
Coexistence of six different attractors projected on the *x*_1_ − *x*_2_ plane. (a) Coexistence of two chaotic attractor and four quasi-periodic attractors when *c* = 0.3. (b) Coexistence of one hyperchaotic attractor starting from *ξ*_1_ (blue) and five quasi-periodic attractors when *c* = 0.85. (c) Coexistence of one chaotic attractor starting from *ξ*_4_ (magenta), one periodic attractor (blue) and four quasi-periodic attractors when *c* = 2.9.

Here the bifurcation diagram of system (1) with respect to parameter *c* is calculated and plotted starting from the six different initial points *ξ*_1_, *ξ*_2_, *ξ*_3_, *ξ*_4_, *ξ*_5_ and *ξ*_6_. Fix *a* = 0.1, *b* = 0.1 and *d* = 0.01, it can be observed from the bifurcation diagram that the new 10-D hyperchaotic system (1) exhibit six different dynamical evolutions when *c* ∈ [0;3], as depicted in Fig 15. When *c* ∈ ([0;0.4], [1.1;2.2]), the new 10-D hyperchaotic system (1) has coexistence of two chaotic and four quasi-periodic attractors as shown in Fig 16(a). Coexistence of one chaotic starting from *ξ*_1_ and five quasi-periodic attractors is determined when *a* ∈ ([0.5;0.7], [2.3;3]), as depicted in Fig 16(b). Finally, when *c* ∈ [0.8;1], we can observe the coexistence of one chaotic attractor starting from *ξ*_4_ and five quasi-periodic attractors as shown if Fig 16(c).

Dynamics, Kaplan-Yorke fractal dimension and Lyapunov exponents for all coexisting attractors when *c* ∈ [0;3], are listed in Table 8.

**Table 8:** Lyapunov exponents, Kaplan-Yorke dimensin and dynamics of system (1) coexisisting attractors with parameter *c* varying.

*c*	*ξ*_*i*_	*LE*_1_	*LE*_2_	*LE*_3_	*LE*_4_	*LE*_5_	*LE*_6_	*LE*_7_	*LE*_8_	*LE*_9_	*LE*_10_	*D*_*KY*_	Dynamics
0.3	*ξ*_1_	0.062	0	0	0	0	0	-0.010	-0.053	-0.098	-0.492	7.981	Chaos
	*ξ*_2_, *ξ*_3_	0	0	0	0	0	0	-0.040	-0.044	-0.203	-0.413	0	Quasi-Periodic
	*ξ*_4_	0.032	0	0	0	0	0	-0.019	-0.045	-0.107	-0.467	7.288	Chaos
	*ξ*_5_, *ξ*_6_	0	0	0	0	0	-0.036	-0.039	-0.086	-0.091	-0.625	0	Quasi-Periodic
0.9	*ξ*_1_	0.129	0.010	0	0	0	0	-0.010	-0.027	-0.101	-0.603	6.52	Hyperchaos
	*ξ*_2_, *ξ*_3_	0	0	0	0	-0.017	-0.021	-0.023	-0.025	-0.069	-0.607	0	Quasi-Periodic
	*ξ*_4_	0	0	0	0	0	-0.004	-0.046	-0.083	-0.103	-0.559	0	Quasi-Periodic
	*ξ*_5_, *ξ*_6_	0	0	0	0	0	-0.021	-0.024	-0.112	-0.117	-0.622	0	Quasi-Periodic
2.9	*ξ*_1_	0	-0.003	-0.006	-0.007	-0.009	-0.010	-0.014	-0.049	-0.052	-0.772	0	Periodic
	*ξ*_2_, *ξ*_3_	0	0	0	-0.005	-0.006	-0.008	-0.012	-0.014	-0.313	-0.364	0	Quasi-Periodic
	*ξ*_4_	0.111	0.011	0	0	0	-0.009	-0.010	-0.017	-0.067	-0.587	9.031	Hyperchaos
	*ξ*_5_, *ξ*_6_	0	0	0	-0.006	-0.007	-0.010	-0.01	1 -0.088	-0.096	-0.679	0	Quasi-Periodic

To the best of the authors knowledge, no research has been done on the new 10-D hyperchaotic chaotic system that exhibiting different coexisting attractors with the variation of its parameters.

## Synchronization of the new 10D hyperchaotic system with a set of chaotic systems

This section study the synchronization of the proposed new 10-D hyperchaotic with three diverse Hyperchaotic and chaotic systems via active controllers. One considers a set of three systems as master system. The slave system will be the new 10D Hyperchaotic system. The idea is to synchronize the first three coordinates of the new 10-D hyperchaotic system with coordinates of the 3D system (17), the second three state coordinates of the new 10-D hyperchaotic system will be synchronized with the state coordinates of the 3D system (18). Finally, we will synchronize the last four coordinates of the new 10-D hyperchaotic system with the coordinates of the 4D hyeprchaotic system (19).

### The first 3-D chaotic system

This subsection review the 3D chaotic system [35], which has six terms with two nonlinearities and it was given by:

$$\begin{array}{c} \left\{ \begin{array}{c} {\dot{x}}_{11}=e\left(x_{12}-x_{11}\right),\\ {\dot{x}}_{12}=x_{1}x_{3},\\ {\dot{x}}_{13}=50-fx_{11}^{4}-gx_{13}. \end{array}\right. \end{array}$$ (17)

**Fig 17:**
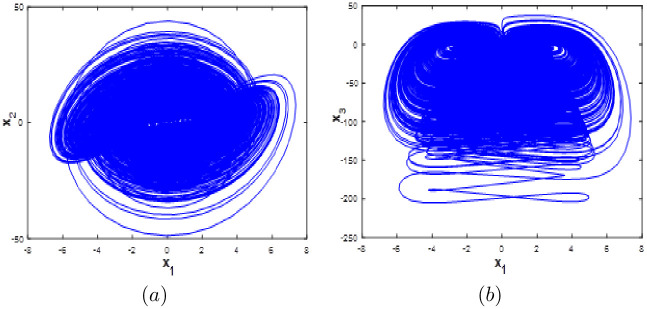
Phase portraits of the hyperchaotic system (17): (a) *x*_1_ − *x*_2_ attractor, (b) *x*_1_ − *x*_3_ attractor.

Suppose the parameters are represented by *e* = 3, *f* = 1 and *g* = 1 and for the initial conditions (0.1; 0.1; 0.1), then, system (17) exhibits a chaotic behaviour with the following values of Lyapunov exponents: *LE*_1_ = 1.386, *LE*_2_ = 0, *LE*_3_ = −5.386 The phase portraits of the 3D chaotic system (17) are depicted in Fig 17.

### The second 3-D chaotic system

This subsection review the 3D chaotic system [36], which has three quadratic nonlinear terms. It was described as follows:

$$\begin{array}{c} \left\{ \begin{array}{c} {\dot{x}}_{21}=x_{22},\\ {\dot{x}}_{22}=hx_{21}x_{23}+kx_{22}^{2}-mx_{2}-x_{22}x_{23},\\ {\dot{x}}_{23}=x_{22}^{2}-1. \end{array}\right. \end{array}$$ (18)

**Fig 18:**
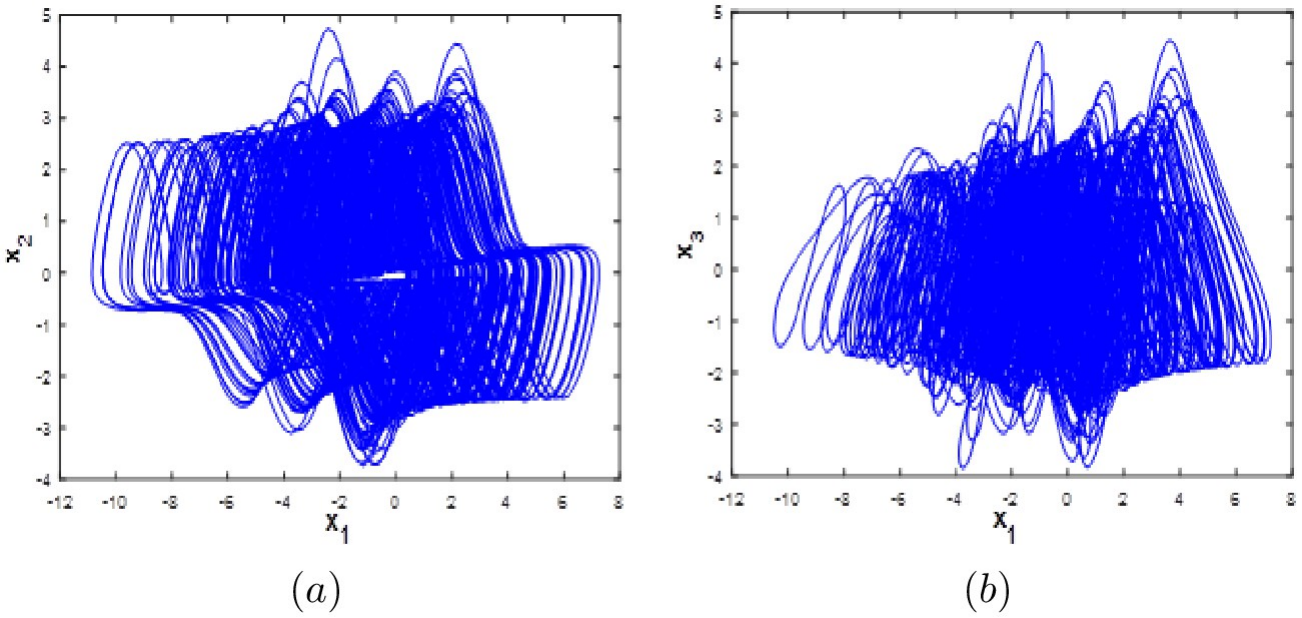
Phase portraits of the hyperchaotic system (18): (a) *x*_1_ − *x*_2_ attractor, (b) *x*_1_ − *x*_3_ attractor.

When the coefficients take the values *h* = 0.1, *k* = 0.1, *m* = 0.15 and for the initial conditions (0.2, 0.2, 0.2), system (18) exhibits a chaotic behaviour with the following values of *LE*_1_ = 0.053, *LE*_2_ = 0, *LE*_3_ = −0.183. The phase portraits of the 3D chaotic system are presented in Fig 18.

### The 4-D hyperchaotic system

This subsection review the 4D hyperchaotic system [37], which has four nonlinear terms and line equilibrium. It was described as follows:

$$\begin{array}{c} \left\{ \begin{array}{c} {\dot{x}}_{31}=x_{32}-x_{31}x_{33}-x_{32}x_{33},\\ {\dot{x}}_{32}=nx_{31}x_{33}+x_{34},\\ {\dot{x}}_{33}=x_{32}^{2}-x_{33}+x_{34},\\ {\dot{x}}_{34}=px_{32}. \end{array}\right. \end{array}$$ (19)

**Fig 19:**
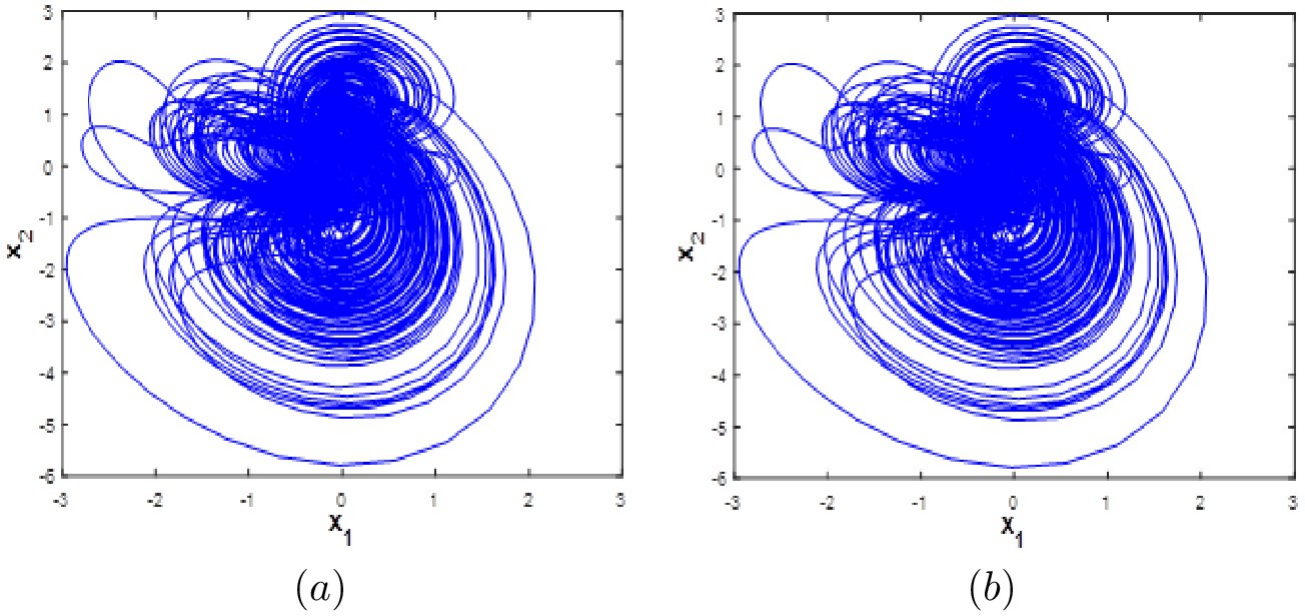
Phase portraits of the hyperchaotic system (19): (a) *x*_1_ − *x*_2_ attractor, (b) *x*_1_ − *x*_3_ attractor.

When the parameters take the values *n* = 3, *p* = −0.08 and for the initial conditions (0.1, 0.1, 0.1, 0.1), system (12) exhibits a hyperchaotic behaviour with the following values of Lyapunov exponents *LE*_1_ = 0.163, *LE*_2_ = 0.024, *LE*_3_ = −1.823. The phase portraits of the 4D hyperchaotic system are shown in Fig 19.

### Design of active controllers for synchronization

Design of active controllers is considered in this subsection, in order to synchronize the new 10-D hyperchaotic system (1) and a set of three multidimensional systems (20). One considers the following set of chaotic systems (The first 3D chaotic system (17), the second 3D chaotic system (18) and the 4D hyperchaotic system (19) as master system:

$$\begin{array}{c} \left\{ \begin{array}{c} {\dot{x}}_{11}=e\left(x_{12}-x_{11}\right),\\ {\dot{x}}_{12}=x_{1}x_{3},\\ {\dot{x}}_{13}=50-fx_{11}^{4}-gx_{13},\\ {\dot{x}}_{21}=x_{22},\\ {\dot{x}}_{22}=hx_{21}x_{23}+kx_{22}^{2}-mx_{2}-x_{22}x_{23},\\ {\dot{x}}_{23}=x_{22}^{2}-1,\\ {\dot{x}}_{31}=x_{32}-x_{31}x_{33}-x_{32}x_{33},\\ {\dot{x}}_{32}=nx_{31}x_{33}+x_{34},\\ {\dot{x}}_{33}=x_{32}^{2}-x_{33}+x_{34},\\ {\dot{x}}_{34}=px_{32}. \end{array}\right. \end{array}$$ (20)

Then, the new 10-D system is studied as a master system and described as follows:

$$\begin{array}{c} \left\{ \begin{array}{c} \dot{x_{1}}=x_{3}+x_{1}x_{2}-x_{1}+u_{1},\\ \dot{x_{2}}=1+a(x_{2}-x_{1}^{4})-x_{1}^{2}+u_{2},\\ \dot{x_{3}}=-x_{1}+x_{3}+x_{4}+u_{3},\\ \dot{x_{4}}=-bx_{3}+cx_{5}+u_{4},\\ \dot{x_{5}}=-x_{4}+x_{6}+u_{5},\\ \dot{x_{6}}=-x_{5}+x_{7}+u_{6},\\ \dot{x_{7}}=-x_{6}+x_{8}+u_{7},\\ \dot{x_{8}}=-x_{7}+(1-d)x_{9}+u_{8},\\ \dot{x_{9}}=-x_{8}+x_{10}+u_{9},\\ {\dot{x}}_{10}=-x_{9}+x_{7}+u_{10}, \end{array}\right. \end{array}$$ (21)

where the functions of the active control to be found is *u*_1_, *u*_2_, *u*_3_, *u*_4_, *u*_5_, *u*_6_, *u*_7_, *u*_8_, *u*_9_ and *u*_10_. The state errors are defined as *e* − 1 = *x*_1_ − *x*_11_, *e*_2_ = *x*_2_ − *x*_12_, *e*_3_ = *x*_3_ − *x*_13_, *e*_4_ = *x*_4_ − *x*_21_, *e*_5_ = *x*_5_ − *x*_2_, *e*_6_ = *x*_6_ − *x*_23_, *e*_7_ = *x*_7_ − *x*_31_, *e*_8_ = *x*_8_ − *x*_32_, *e*_9_ = *x*_9_ − *x*_33_, *e*_10_ = *x*_10_ − *x*_34_ From the slave system (21), we subtract master system (20) including the control functions, thus, we obtain error system as follows:

$$\begin{array}{c} \left\{ \begin{array}{c} \dot{e_{1}}=\dot{x_{1}}-\dot{x_{11}}=x_{3}+x_{1}x_{2}-x_{1}-e\left(x_{12}-x_{11}\right)+u_{1},\\ \dot{e_{2}}=\dot{x_{2}}-\dot{x_{12}}=1+a(x_{2}-x_{1}^{4})-x_{1}^{2}-x_{1}x_{3}+u_{2},\\ \dot{e_{3}}=\dot{x_{3}}-\dot{x_{13}}=-x_{1}+x_{3}+x_{4}-50+fx_{11}^{4}+gx_{13}+u_{3},\\ \dot{e_{4}}=\dot{x_{4}}-\dot{x_{21}}=-bx_{3}+cx_{5}-x_{22}+u_{4},\\ \dot{e_{5}}=\dot{x_{5}}-\dot{x_{22}}=-x_{4}+x_{6}-hx_{21}x_{23}-kx_{22}^{2}+mx_{2}+x_{22}x_{23}+u_{5},\\ \dot{e_{6}}=\dot{x_{6}}-\dot{x_{23}}=-x_{5}+x_{7}-x_{22}^{2}+1+u_{6},\\ \dot{e_{7}}=\dot{x_{7}}-\dot{x_{31}}=-x_{6}+x_{8}-x_{32}+x_{31}x_{33}+x_{32}x_{33}+u_{7},\\ \dot{e_{8}}=\dot{x_{8}}-\dot{x_{32}}=-x_{7}+(1-d)x_{9}-nx_{31}x_{33}-x_{34}+u_{8},\\ \dot{e_{9}}=\dot{x_{9}}-\dot{x_{33}}=-x_{8}+x_{10}-x_{32}^{2}+x_{33}-x_{34}+u_{9},\\ {\dot{e}}_{10}=\dot{x_{10}}-\dot{x_{34}}=-x_{9}+x_{7}-px_{32}+u_{10}. \end{array}\right. \end{array}$$ (22)

Our aim is to design the active control functions, which control the error system to be asymptotically stable; in order to ascertain synchronization between the new 10-D hyperchaotic system (21) and set of systems (20).

By choosing the active control functions as the follows:

$$\begin{array}{c} \left\{ \begin{array}{c} u_{1}=e\left(x_{12}-x_{11}\right)-x_{3}-x_{1}x_{2}-e_{1},\\ u_{2}=x_{1}x_{3}-1-a\left(x_{2}-x_{1}^{4}\right)+x_{1}^{2}-e_{2},\\ u_{3}=50-fx_{11}^{4}-gx_{13}+x_{1}-x_{3}-x_{4}-e_{3},\\ u_{4}=x_{22}+bx_{3}-cx_{5}+bx_{3}-cx_{5}-e_{4},\\ u_{5}=hx_{21}x_{23}+kx_{22}^{2}-mx_{2}-x_{22}x_{23}+x_{4}-x_{6}-e_{5},\\ u_{6}=x_{22}^{2}-1+x_{5}-x_{7}-e_{6},\\ u_{7}=x_{32}-x_{31}x_{33}-x_{32}x_{33}+x_{6}-x_{8}-e_{7},\\ u_{8}=nx_{31}x_{33}+x_{34}+x_{7}-(1-d)x_{9}-e_{8},\\ u_{9}=x_{32}^{2}-x_{33}+x_{34}+x_{8}-x_{10}-e_{9},\\ u_{10}=px_{32}+x_{9}-x_{7}-e_{10}. \end{array}\right. \end{array}$$ (23)

The dynamical equations of error system becomes:

$$\begin{array}{c} \left\{ \begin{array}{c} \dot{e_{1}}=-e_{1},\\ \dot{e_{2}}=-e_{2},\\ \dot{e_{3}}=-e_{3},\\ \dot{e_{4}}=-e_{4},\\ \dot{e_{5}}=-e_{5},\\ \dot{e_{6}}=-e_{6},\\ \dot{e_{7}}=-e_{7},\\ \dot{e_{8}}=-e_{8},\\ \dot{e_{9}}=-e_{9},\\ {\dot{e}}_{10}=-e_{10}. \end{array}\right. \end{array}$$ (24)

It can be noted from (24) that after applying the proposed active control functions (23) the error system becomes linear with the following state representation:

$$\begin{array}{c} \left[\begin{array}{c} {\dot{e}}_{1}\\ {\dot{e}}_{2}\\ {\dot{e}}_{3}\\ {\dot{e}}_{4}\\ {\dot{e}}_{5}\\ {\dot{e}}_{6}\\ {\dot{e}}_{7}\\ {\dot{e}}_{8}\\ {\dot{e}}_{9}\\ {\dot{e}}_{10} \end{array}]=[\begin{array}{cccccccccc} -1 & 0 & 0 & 0 & 0 & 0 & 0 & 0 & 0 & 0\\ 0 & -1 & 0 & 0 & 0 & 0 & 0 & 0 & 0 & 0\\ 0 & 0 & -1 & 0 & 0 & 0 & 0 & 0 & 0 & 0\\ 0 & 0 & 0 & -1 & 0 & 0 & 0 & 0 & 0 & 0\\ 0 & 0 & 0 & 0 & -1 & 0 & 0 & 0 & 0 & 0\\ 0 & 0 & 0 & 0 & 0 & -1 & 0 & 0 & 0 & 0\\ 0 & 0 & 0 & 0 & 0 & 0 & -1 & 0 & 0 & 0\\ 0 & 0 & 0 & 0 & 0 & 0 & 0 & -1 & 0 & 0\\ 0 & 0 & 0 & 0 & 0 & 0 & 0 & 0 & -1 & 0\\ 0 & 0 & 0 & 0 & 0 & 0 & 1 & 0 & 0 & -1 \end{array}][\begin{array}{c} e_{1}\\ e_{2}\\ e_{3}\\ e_{4}\\ e_{5}\\ e_{6}\\ e_{7}\\ e_{8}\\ e_{9}\\ e_{10} \end{array}\right] \end{array}$$ (25)

It is easy to check that all eigenvalues of the states matrix (25) are negatives, so, based on Routh-Hurwitz condition; the error system is stable which assure synchronization between the slave system (21) and master system (20). So, the designed active functions ensure that the first three states of the 10 D system will be synchronized with the states of the first chaotic system (17). The second three states of the new 10-D hyperchaotic system will be synchronized with the states of second chaotic system (18) and the last four states of the new 10-D hyperchaotic system will be synchronized with the states of the 4D hyperchaotic system (19).

## Simulation results

**Fig 20:**
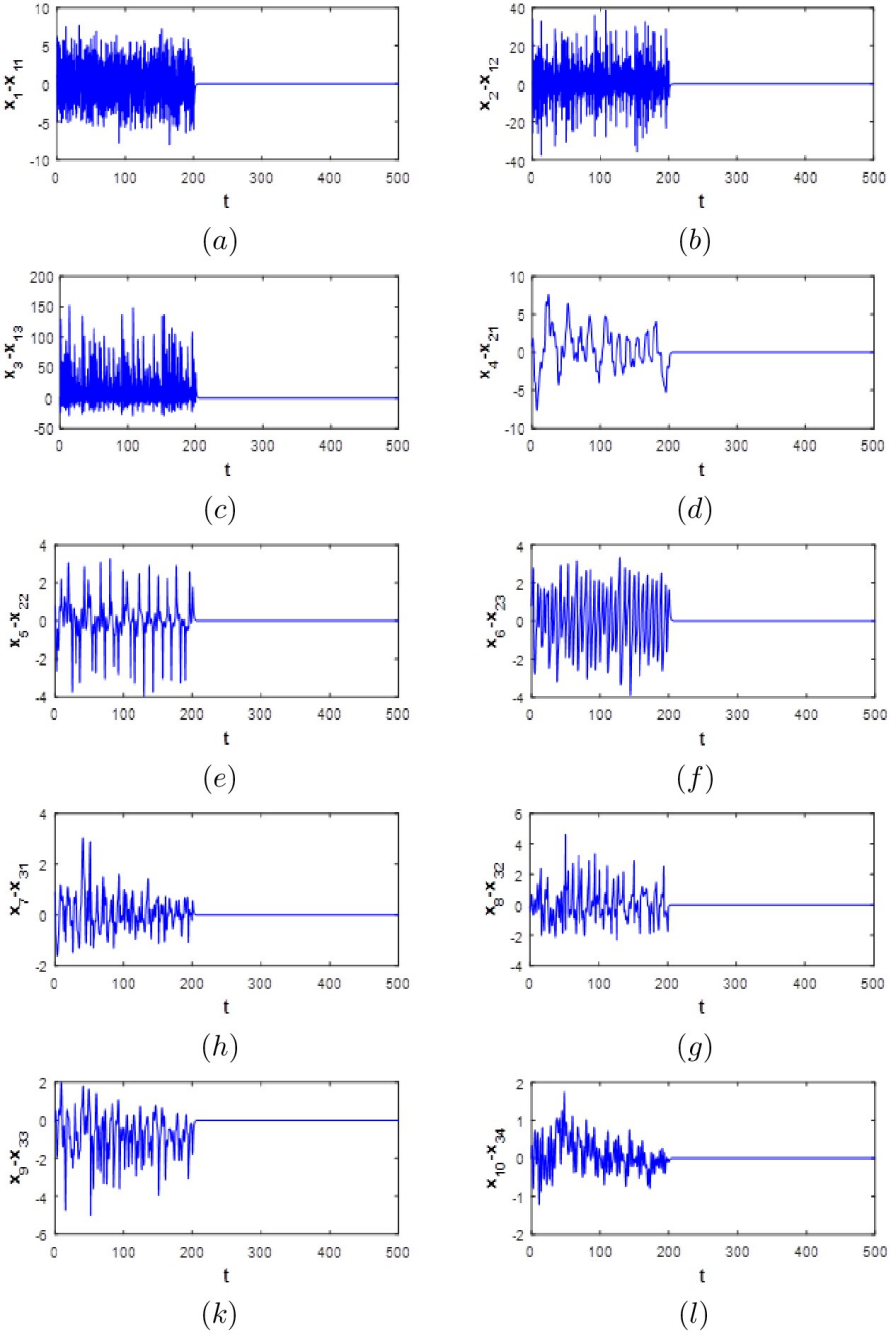
Time evolution of the synchronization errors with controllers deactivated (*t* < 200*s*) and activated (*t* > 200*s*).

For numerical simulations the initial conditions of the master system (10) are chosen as: (0.1, 0.1, 0.1, 0.2, 0.2, 0.2, 0.1, 0.1, 0.1, 0.1) The parameters of the master system are chosen as: *e* = 3, *f* = 1, *g* = 1, *h* = 0., *k* = 0.1, *m* = 0.15, *n* = 3 and *p* = −0.08 The initial conditions of the slave system (10) are chosen as (1, 1, 1, 1, 1, 1, 1, 0, 0, 0) The parameters of the slave system are chosen as: *a* = 0.1, *b* = 0.1, *c* = 1.8 and *d* = 0. Active controllers is switched on at *t* = 200*s* and all the states error time evolution are depicted in Fig 20. The results shows that all the ten states of error system (22) evolve chaotically with time when the active controllers are deactivated (when *t* < 200*s*) indicating non synchronization. After that (when *t* ≥ 200*s*), the controllers are activated and it can be seen that all the states synchronization error converge rapidly to zero. So, simulation results showing the success of the proposed active controllers (23) to synchronize the new 10D hyperchaotic system with a class of three multidimensional chaotic and hyperchaotic systems. To the best of the authors knowledge, no study has been done to investigates the synchronization of the new 10D hyperchaotic system with the active control strategy. Also, synchronization of the proposed 10D system (1) with a class of low dimensional systems making it very desirable to use in secure communications schemes that need high complexity.

## Circuit implementation of the new 10-D system

**Fig 21:**
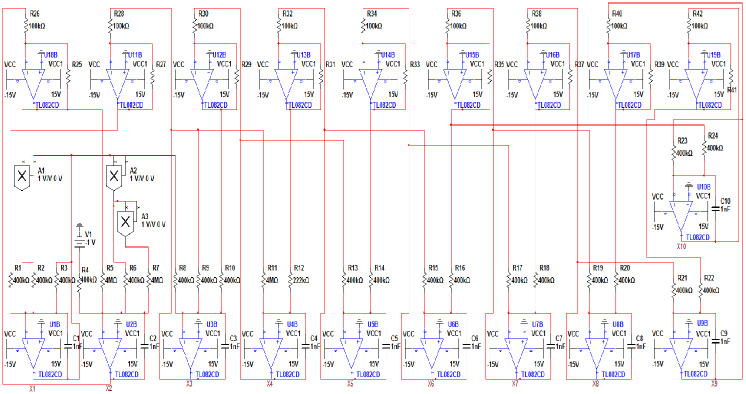
Electronic circuit schematic of the proposed 10-D hyperchaotic system (1).

In order to test system (1) physical feasibility, an equivalent electronic circuit for the new 10-D hyperchaotic system (1) is developed using Multisim 13.0 software as depicted in Fig 21. Using the Kirchhoff’s laws to the circuit in Fig 21, the circuital equations of the new 10D Hyperchaotic system (1) becomes:

$$\begin{array}{c} \left\{ \begin{array}{c} {\dot{x}}_{1}=\frac{1}{R_{1}C_{1}}x_{3}-\frac{1}{R_{2}C_{1}}x_{1}x_{2}-\frac{1}{R_{3}C_{1}}x_{1},\\ {\dot{x}}_{2}=\frac{1}{R_{4}C_{2}}V+\frac{1}{R_{5}C_{2}}x_{2}-\frac{1}{R_{6}C_{2}}x_{1}^{2}-\frac{1}{R_{7}C_{2}}x_{1}^{4},\\ {\dot{x}}_{3}=-\frac{1}{R_{8}C_{3}}x_{1}-\frac{1}{R_{9}C_{3}}x_{3}+\frac{1}{R_{10}C_{3}}x_{4},\\ {\dot{x}}_{4}=-\frac{1}{R_{11}C_{4}}x_{3}+\frac{1}{R_{12}C_{4}}x_{5},\\ {\dot{x}}_{5}=-\frac{1}{R_{13}C_{5}}x_{4}+\frac{1}{R_{14}C_{5}}x_{6},\\ {\dot{x}}_{6}=-\frac{1}{R_{15}C_{6}}x_{5}+\frac{1}{R_{16}C_{6}}x_{7},\\ {\dot{x}}_{7}=-\frac{1}{R_{17}C_{7}}x_{6}+\frac{1}{R_{18}C_{7}}x_{8},\\ {\dot{x}}_{8}=-\frac{1}{R_{19}C_{8}}x_{7}+\frac{1}{R_{20}C_{8}}x_{9},\\ {\dot{x}}_{9}=-\frac{1}{R_{21}C_{9}}x_{8}+\frac{1}{R_{22}C_{9}}x_{10},\\ {\dot{x}}_{10}=-\frac{1}{R_{23}C_{10}}x_{9}+\frac{1}{R_{27}C_{10}}x_{7}. \end{array}\right. \end{array}$$ (26)

By using TL082CD operational amplifiers, the circuit would have nine reversers and ten integrators, also this study considered three multipliers IC AD633. All active devices have a supply voltage ±15*V*. The values of circuital components are selected as follows: *R*_1_ = *R*_2_ = *R*_3_ = *R*_4_ = *R*_6_ = *R*_8_ = *R*_9_ = *R*_10_ = 400*K*Ω, *R*_5_ = *R*_7_ = *R*_11_ = 4*M*Ω, *R*_12_ = 222.23*K*Ω, *R*_20_ = 404.04*K*Ω, *R*_*i*_ = 400*K*Ω for *i* = 13, …, 19 and *i* = 21, …, 24, *R*_*i*_ = 100*K*Ω for *i* = 25, …, 42, and *C*_*i*_ = 1*nf* for *i* = 1, …, 10.

**Fig 22:**
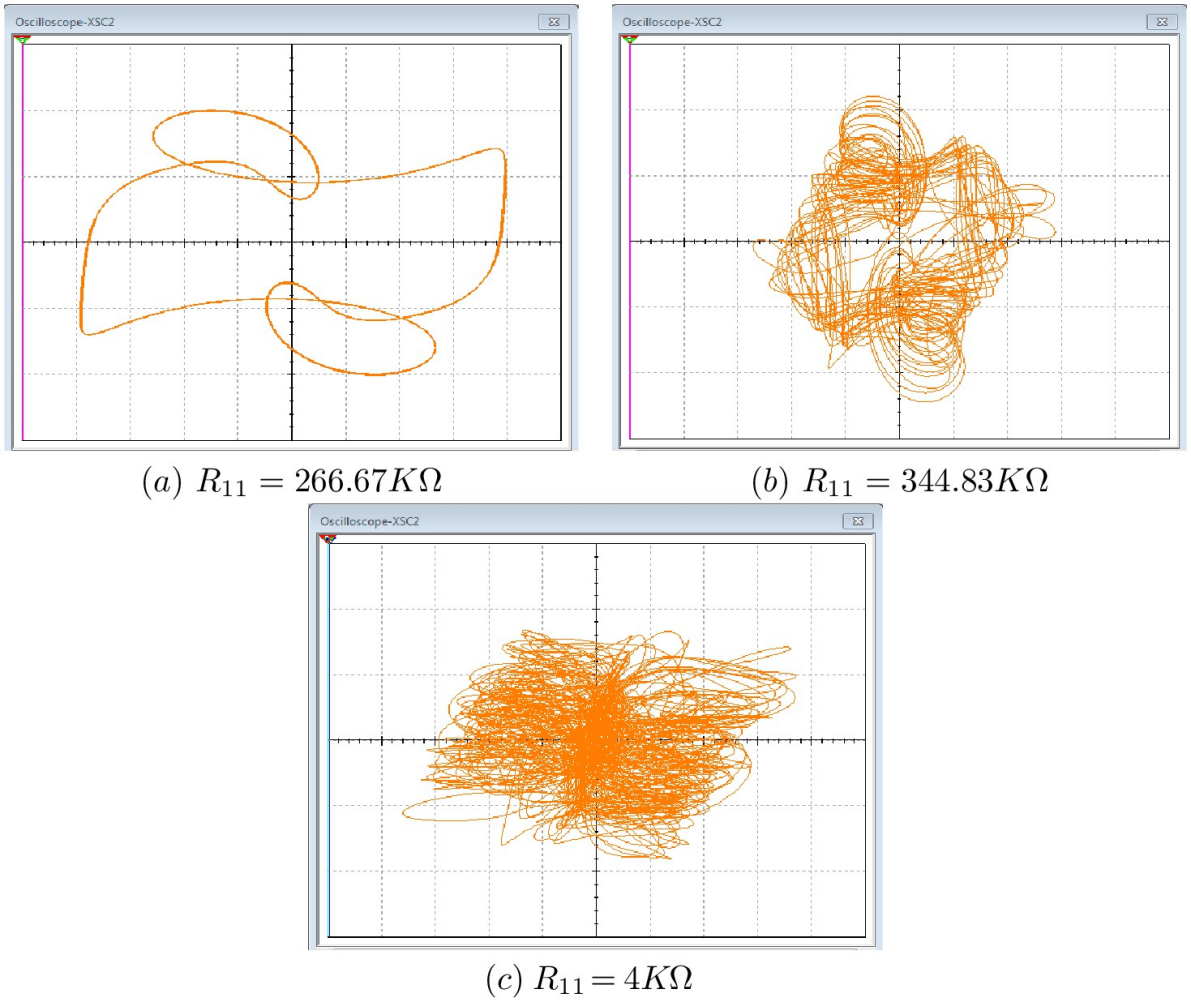
Experimental phase portraits of the system (1) *x*_3_ − *x*_4_ plane. (a): Periodic orbit, (b): Chaotic attractor and (c): Hyperchaotic attractor.

Fig 22(a)–22(c) shows the periodic attractor, the chaotic attractor and the hyperchaotic attractor respectively derived by Multisim 13.0. It can be noticed that the Multisim 13.0 simulation results are akin to the Matlab results depicted in Fig 6(a)–6(c) which confirm the proposed 10-D system (1) physical feasibility.

**Fig 23:**
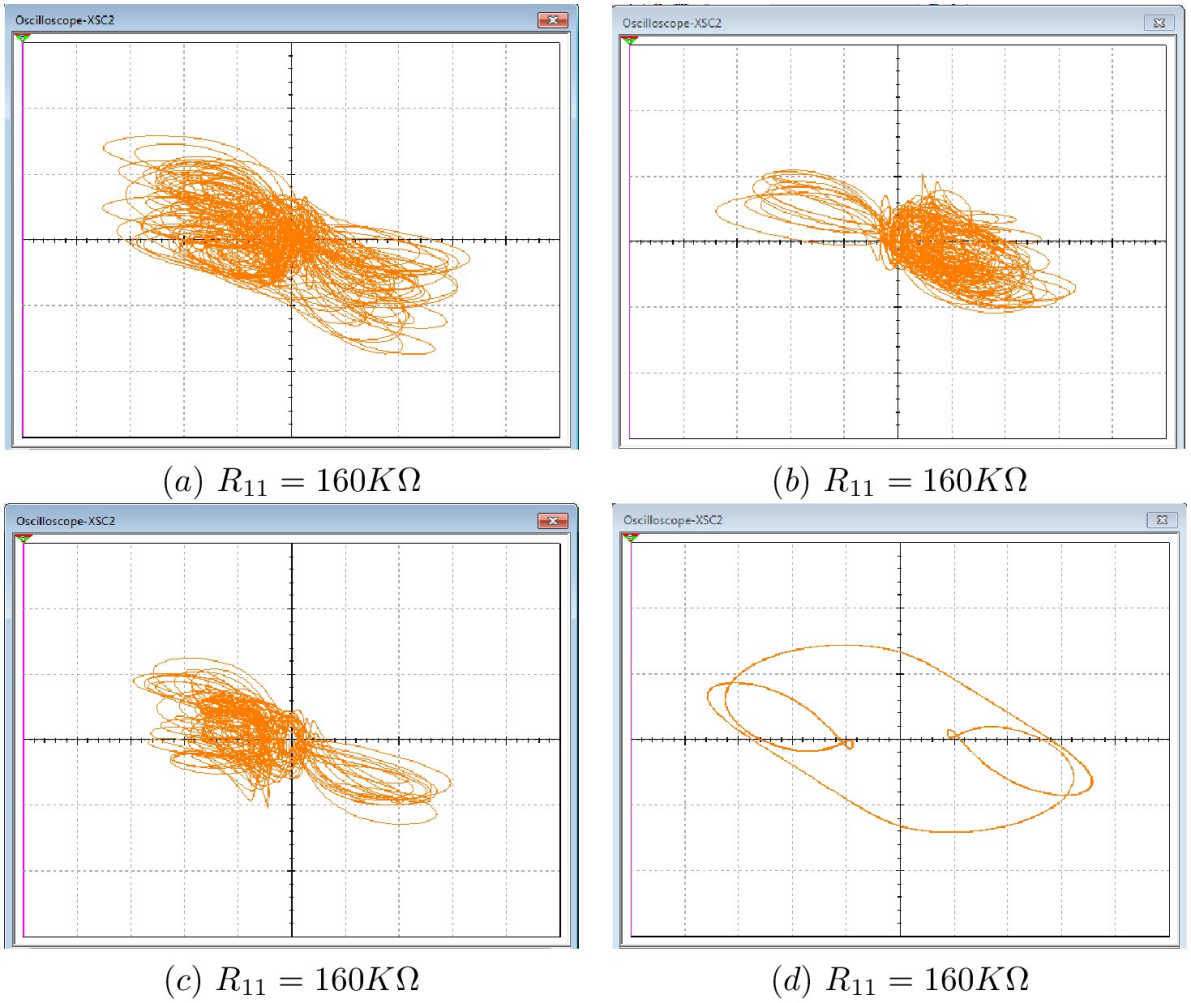
Experimental phase portraits of four coexisting attractors: (a), (b) and (c) three chaotic attractors with initial points (0,0,0,0,0,0,0,0,0,0.5), (0,0,0,0,0,0,0,0,0,±1); (d) periodic attractor with initial point (1,0,0,0,0,0,0,0,0,0).

Fig 23 shows four coexistence attractors obtained from the Multisim 13.0 based implementation of the 10-D system (1) for similar values of coefficients *a* = *b* = 0.1, *c* = 1.8, *d* = 0.01 and four different initial conditions *ξ*_1_, *ξ*_2_, *ξ*_3_ and *ξ*_4_

Fig 23(a)–23(d) shows respectively good compatibility with the four coexisting attractors (the magenta chaotic attractor, the red chaotic attractor, the green chaotic attractor and the blue periodic attractor) depicted in Fig 14(c) using Matlab software. These results confirm the physical existence of the coexisting attractors in the proposed 10-D system (1).

## Conclusion

In this work, a new ten-dimensional hyperchaotic system is first presented; the new system contains four positive parameters and twenty-three terms with two quadratic and a quartic nonlinearities. The new system has many specifics properties, it has three unstable equilibrium points, it can exhibits four different dynamical behaviours (periodic, quasi-periodic, chaos and hyperchaos) for special values of parameters. In addition, the new system may generate many coexisting attractors with high fractal dimension when fixing the parameters and changing the initial conditions. Dynamical properties of the new system is investigated using Lyapunov exponents, Kaplan-Yorke dimension, bifurcation diagrams, phase portraits, equilibrium points stability and dissipativity. The idea of synchronizing the new 10-D high dimensional system with a set of three low dimensional system is applied by using active controllers; which guarantee the convergence of the synchronization errors to zero asymptotically. Finally, in order to prove the real feasibility of the new system and the physical existence of the coexisting attractor, an equivalent electronic circuit was designed using Multisim. The obtained results show a good agreement with Matlab results, which confirm the feasibility of both the 10-D system and its dynamical behaviours. We strongly believe that the new 10-D Hyperchaotic system with its high dimension, very complex dynamic and easy to implement circuit schematic can be applied in various chaotic-based applications. The hardware implementations of the new systems along with their applications are considered as the future direction of the work.
